# Inhibition potential of natural flavonoids against selected omicron (B.1.19) mutations in the spike receptor binding domain of SARS-CoV-2: a molecular modeling approach

**DOI:** 10.1080/07391102.2023.2291165

**Published:** 2023-12-19

**Authors:** Anuj Kumar, Mansi Dutt, Budheswar Dehury, Gustavo Sganzerla Martinez, Cynthia L. Swan, Alyson A. Kelvin, Christopher D. Richardson, David J. Kelvin

**Affiliations:** a Laboratory of Immunity, Shantou University Medical College, Shantou, China; b Department of Microbiology and Immunology, Faculty of Medicine, Dalhousie University, Halifax, Canada; c Department of Paediatrics, IWK Health Center, Canadian Centre for Vaccinology (CCfV), Halifax, Canada; d Bioinformatics Division, ICMR-Regional Medical Research Centre, Bhubaneswar, India; e Vaccine and Infectious Disease Organization (VIDO), University of Saskatchewan, Saskatoon, Canada; f Department of Biochemistry, Microbiology, and Immunology, University of Saskatchewan, Saskatoon, Canada

**Keywords:** SARS-CoV-2, omicron (B.1.19), receptor binding domain (RBD), natural flavonoids, molecular dynamics (MD) simulations

## Abstract

The omicron (B.1.19) variant of contagious severe acute respiratory syndrome coronavirus 2 (SARS-CoV-2), is considered a variant of concern (VOC) due to its increased transmissibility and highly infectious nature. The spike receptor-binding domain (RBD) is a hotspot of mutations and is regarded as a prominent target for screening drug candidates owing to its crucial role in viral entry and immune evasion. To date, no effective therapy or antivirals have been reported; therefore, there is an urgent need for rapid screening of antivirals. An extensive molecular modelling study has been performed with the primary goal to assess the inhibition potential of natural flavonoids as inhibitors against RBD from a manually curated library. Out of 40 natural flavonoids, five natural flavonoids, namely tomentin A (−8.7 kcal/mol), tomentin C (−8.6 kcal/mol), hyperoside (−8.4 kcal/mol), catechin gallate (−8.3 kcal/mol), and corylifol A (−8.2 kcal/mol), have been considered as the top-ranked compounds based on their binding affinity and molecular interaction profiling. The state-of-the-art molecular dynamics (MD) simulations of these top-ranked compounds in complex with RBD exhibited stable dynamics and structural compactness patterns on 200 nanoseconds. Additionally, complexes of these molecules demonstrated favorable free binding energies and affirmed the docking and simulation results. Moreover, the post-simulation validation of these interacted flavonoids using principal component analysis (PCA) revealed stable interaction patterns with RBD. The integrated results suggest that tomentin A, tomentin C, hyperoside, catechin gallate, and corylifol A might be effective against the emerging variants of SARS-CoV-2 and should be further evaluated using *in-vitro* and *in-vivo* experiments.

Communicated by Ramaswamy H. Sarma

## 1. Introduction

Severe acute respiratory syndrome coronavirus-2 **(**SARS-CoV-2) has caused the unprecedented viral pandemic of coronavirus disease (COVID-19) which led to a worldwide health emergency that caused global economic and social disruption. The first case of this contagious disease was officially recorded in Wuhan, Hubei Province, China, and quickly spread around the globe (Huang et al., 2020; Mackenzie & Smith, 2020). Based on the infection and high mortality rates in immunocompromised patients, the World Health Organization (WHO) declared this viral disease a Public Health Emergency of International Concern (PHEIC) on 30 January 2020 and announced it to be a pandemic on 11 March 2020 (Shi et al., 2020; Wilder-Smith & Osman, 2020; Yu et al., 2020). As of 19 April 2023, COVID-19, caused by the SARS-CoV-2 virus, has been responsible for more than 763 million confirmed cases globally, and it is estimated that over 20 million deaths have resulted from the disease (Msemburi et al., 2022; Van Noorden, 2022) (https://covid19.who.int/). Despite widespread vaccination programs against SARS-CoV-2, the continued emergence of new variants has caused panic responses in different countries and territories around the world. These variants have been classified into three major classes based on their mutations and infection patterns, including variants of interest (VOIs), variants of concern (VOCs), and variants of high consequences (VOHc) (Karim & Karim, 2021). The Omicron (B.1.19) variant has been recognized as a VOC based on its high transmission rate and number of mutations. Compared with any previous variant, this variant spreads faster but causes less severe symptoms (Ren et al., 2022). Per the WHO website, the first omicron case originated in South Africa on 24 November 2021 (https://www.who.int/). It has been well documented that the omicron variant reduces antibody neutralization and affects the treatment response in a significant manner (Hakami, 2022; Hewins et al., 2022). The most common symptoms of this variant experienced among infected patients include headache, body aches, muscle aches, cough, fever, generalized myalgia, and fatigue (Arjun et al., 2022; Hewins et al., 2022). The spike (S) protein of the SARS-CoV-2 plays a principal role in viral attachment, fusion, and entry (Tai et al. 2020). Due to its indispensable functions, the S protein has been defined as one of the important targets for vaccine development, antibody-blocking therapy, and screening of small molecules as potential inhibitors (Huang et al., 2020; Tai et al. 2020). This prominent drug target is comprised of two subunits, S1 and S2. The S1 subunit contains a receptor-binding domain (RBD, 319–541 residues) (Huang et al., 2020). The conserved RBD is well known to recognize and bind to the host receptor angiotensin-converting enzyme 2 (ACE2) (Huang et al., 2020). The interaction between the RBD and ACE2 is important for the first step of viral infection (Ni et al., 2019). ACE2 is a homolog of angiotensin-converting enzyme (ACE), containing 805 amino acids (Shirbhate et al., 2020). This protein functions as an enzyme (carboxypeptidase) and is known to degrade the angiotensin II peptides on the cell membrane surface (Kuba et al., 2021). ACE2 is generally found on the surface of cells in different tissues (brain, heart, kidney, lung, and testis) and is defined as the decisive target for the SARS-CoV-2 (Ni et al., 2019; Shirbhate et al., 2020).

The omicron variant possesses 50 mutations and a large number of mutations (over 30) have been reported in the S protein that differentiates it from the SARS-CoV-2 wild-type (WT) (Arjun et al., 2022; Hewins et al., 2022; Ren et al., 2022; Sharma et al., 2022). The RBD of the omicron variant harbours 15 key mutations including G339D, S371L, S373P, S375F, N440K, G446S, T478K, G496S, Q498R, K417N, S477N, E484A, Q493R, N501Y, and Y505H. These 15 mutations in the RBD increase the affinity of the S protein for the ACE2 receptor and show the high transmissibility of the omicron variant in comparison with other variants of SARS-CoV-2 (da Costa et al., 2022; Tian et al., 2021). Several studies have probed the role of these mutations and reported that these mutations play a principal role in the stronger binding of the S protein to the ACE2 receptor and accelerate the infection with mild symptoms (da Costa et al., 2022; Tian et al., 2021). The N501Y mutation is exclusively found in different variants including B.1.1.7 (alpha), B.1.351 (beta), P.1 (gamma), and B.1.19 (omicron). RBD with this key mutation strengthens its binding to receptor ACE2, leading to a higher rate of transmission of SARS-CoV-2 variants (Tian et al., 2021). Despite the availability of many bivalent vaccines in the market, there is still a need for more effective methods for the treatment of emerging variants including omicron; therefore, small molecules-based therapeutics are urgently required to cope with omicron and other variants. Recently, several studies have reported the inhibition potential of different classes of natural compounds against RBD of the omicron variant using an *in-silico* approach (Bahadur Gurung et al., 2022; Goc et al., 2022; Hakami, 2022; Khan et al., 2022; Parihar et al., 2022; Sharanya CS et al., 2022; Tarek Ibrahim & Tao, 2023; Tuli et al., 2022). Flavonoids are an important class of phytochemicals with variable phenolic structures; they occur ubiquitously in fruits, vegetables, bark, roots, flowers, stems, legumes, chocolate, and beverages such as tea and red wine (Cui et al., 2016; Panche et al., 2016). More than 6,000 flavonoid molecules have been structurally identified from natural sources. Flavonoids have been classified into different sub-classes based on their structural complexity including anthocyanidins, flavan-3-ols, flavonols, flavones, flavanones, and isoflavones (Hui et al., 2013). These phenolic plant compounds are well identified in traditional and modern medicines in the treatment and prevention of several diseases including type 2 diabetes mellitus; cardiovascular disease; cancer; neuroprotection and cognitive function; hypertension; and viral infection (Cui et al., 2016; Kumar et al., 2018; Kumar & Pandey, 2013; Panche et al., 2016; Taheri et al., 2020; Ullah et al., 2020). As antivirals, several flavonoids have been reported to inhibit different drug targets of COVID-19 (Alzaabi et al., 2022; Ghidoli et al., 2021; Kumar et al., 2021; Russo et al., 2020; Saeedi-Boroujeni & Mahmoudian-Sani, 2021). To date, few *in-silico* studies have been performed to evaluate the inhibition potential of natural flavonoids against RBD of the omicron variant (Hakami, 2022; Hassan et al., 2022). A recent study (Hakami, 2022) investigated the potential of database-derived natural compounds from South African plants against the RBD of omicron. This study reported four natural flavonoids, amentoflavone, 1,2,3,6-tetragalloylglucose, luteolin, and quercetin, as potential inhibitors against RBD of the S protein from the omicron variant of SARS-CoV-2 using an integrated molecular docking and MD simulations approach. The molecular docking investigation of amentoflavone, 1,2,3,6-tetragalloylglucose, luteolin, and quercetin with RBD of omicron demonstrated the binding energies of −8.41, −9.35, −6.99, and −6.93 kcal/mol, respectively (Hakami, 2022). In a very recent study, Hassan et al., 2022, investigated the molecular interaction patterns of fifteen flavonoids derived from *Echium angustifolium* and *Prunus persica* against RBD of omicron. Based on their binding affinities and MD simulations followed by MM/PBSA analysis, three flavonoids, specifically eshiumin A, kaempferol 3 − O−(3”−O − *α*−rhamnopyranosyl)−α − rhamnopyranoside, and kaempferol 3 −O − *α*−rhamnopyranosyl−(1→6)−*β*−glucopyranosyl−5−O−*α*−rhamnopyranoside, were reported as the top-ranked molecules against the binding site of omicron. Despite the rich antiviral properties and proven inhibition potential against reference SARS-CoV-2, flavonoids exclusively found in foods have not yet been extensively investigated against omicron. New advances in molecular docking and modelling methods have accelerated the efforts of virologists and bioinformaticians to screen the small natural compounds against emerging infectious diseases (Shojapour and Farahmand, 2022). In the present study, we performed virtual screening against the RBD of omicron (B.1.19) and assessed the binding affinity of natural flavonoids from a manually curated library of 40 molecules. Based on their docking and drug-likeness properties, five natural flavonoids, namely, tomentin A, tomentin C, hyperoside, catechin gallate, and corylifol A, were considered the top-ranked drug candidates. Complexes of these selected molecules with RBD were further subjected to molecular dynamics simulations (MDS) on 200 ns to evaluate their stability at the atomic level, followed by molecular mechanics/Poisson–Boltzmann surface area (MM/PBSA) free energy calculations.

## 2. Materials and methods

**Figure 1.**
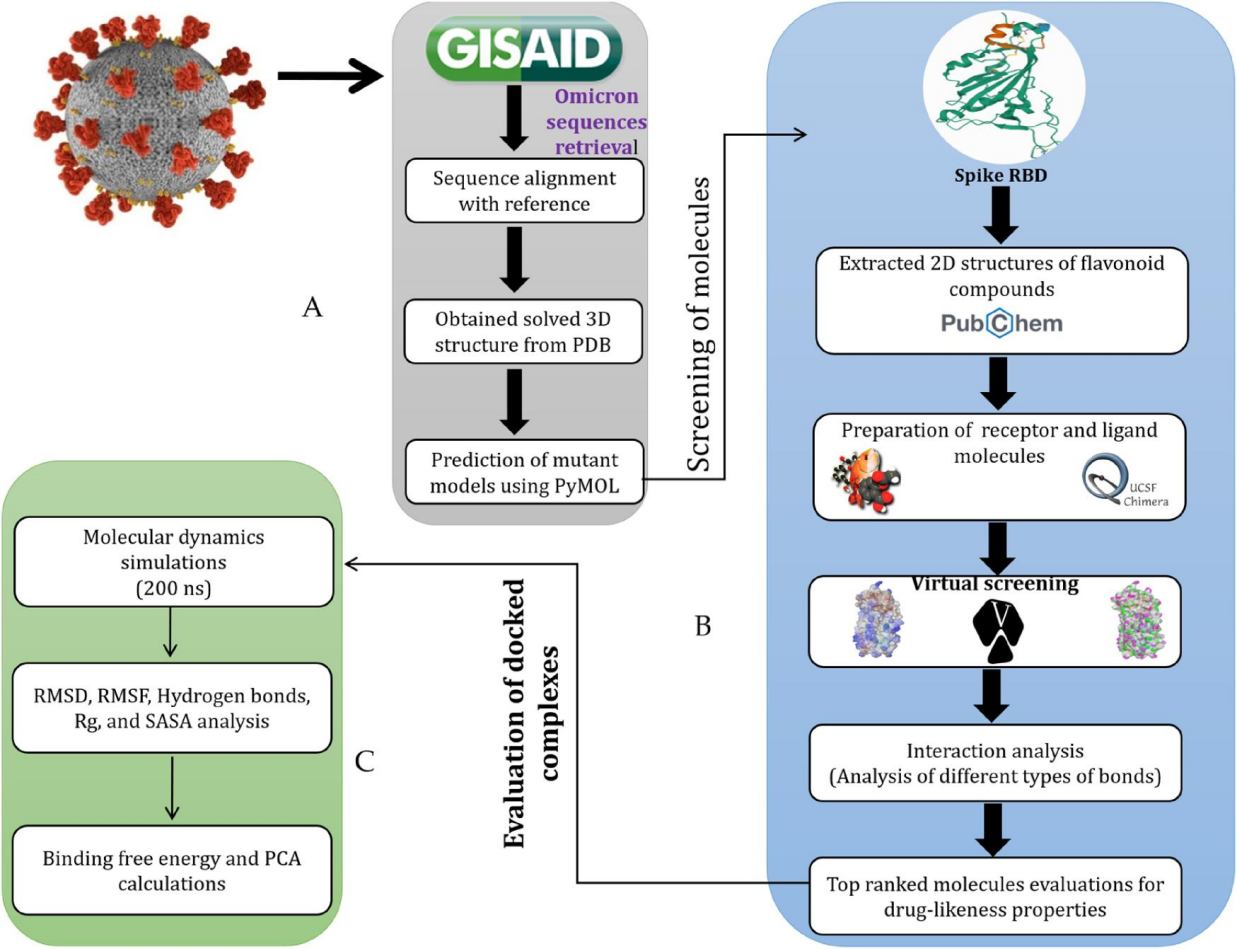
Stepwise representation of pipeline utilized in the present study to identify the natural flavonoids as potential inhibitors against RBD of omicron using an integrated molecular modelling approach. (A) Steps involved in omicron sequence retrieval from GISAID database and extraction of S protein 3D structure from protein Data Bank (PDB) followed by mutant model prediction using PyMOL. (B) Flow of steps utilized in preparation of 3D structure model of omicron RBD and ligand molecules, virtual screening based molecular docking, and molecular interactions analysis followed by drug-likeness properties. (C) Molecular dynamics simulations, binding free energy, and PCA based stability evaluation of docked complexes on 200 ns.

A flowchart reflecting the key steps involved in the screening of flavonoids against RBD of omicron is presented in Figure 1.

### 2.1. Protein structure retrieval and preparation

Linear amino acid sequences of the omicron S protein were obtained from the GISAID database (https://gisaid.org/) in fasta format. To map the key mutations in the RBD of S protein, pairwise sequence alignment was conducted between the SARS-CoV-2 original B-lineage virus and omicron using the BLASTP program (Altschul et al., 1997) available on the National Center for Biotechnology Information (NCBI) (www.ncbi.nlm.nih.gov). Cryo-EM structure of the SARS-CoV-2 omicron S protein in complex with a 2-acetamido-2-deoxy-beta-D-glucopyranose molecule [(Electron Microscopy Data Bank ID: 25759); (PDB ID: 7T9J), at a resolution of 2.79 Å), was attained from the RCSB Protein Data Bank (www.rcsb.org) in PDB format (Berman, 2000; Berman et al., 2000). The selected protein structure was processed using the different protein preparation modules available in AutoDockTools v. 1.5.6 (Morris et al., 2009), UCSF Chimera v. 1.16 (Pettersen et al., 2004), and Discovery Studio 2021 (https://www.3ds.com/products-services/biovia/). Briefly, NAG ligand and different chains (except RBD), water, and hetero atoms were removed and missing hydrogen atoms were added to the structure. Kollman charges were also assigned to the RBD structure. Amber force field ff14SB available in UCSF Chimera was employed to optimize and minimize the structure with a convergence criterion of 0.3 Å root mean square deviation for all the heavy atoms. The clean geometry algorithm of Discovery Studio (https://www.3ds.com/products-services/biovia/) program was applied to side chain correction.

### 2.2. Flavonoids library preparation

In the search for a potential inhibitor against RBD of omicron, an extensive literature survey was conducted to prepare a library of natural flavonoids. As many as 40 natural flavonoid compounds with preclinical or clinical trial evidence against different drug targets (S protein, envelope protein, membrane protein, nucleocapsid, and the main protease) of the SARS-CoV-2 original B-lineage virus were selected for this study (Alzaabi et al., 2022; Ghidoli et al., 2021; Liskova et al., 2021; Russo et al., 2020; Santana et al., 2021; Xu et al., 2020). 3D conformations of all these compounds were downloaded from the comprehensive PubChem database (Kim et al., 2020) in Structure Data File (SDF) format. Merck molecular force field 94 (MMFF94) embedded in the Open Babel package was applied to optimize each molecule of the library followed by the addition of polar hydrogens and Gasteiger charges. Optimized structures of the molecules were converted into the PDBQT format using Open Babel as previously described by Kumar et al., (2022), Kumar, Mishra et al. (2021).

### 2.3. Virtual screening

Site-specific molecular docking was performed to predict the molecular interactions between RBD of omicron and the prepared library of 40 natural flavonoids. AutoDock Vina program was executed on a Linux based workstation with the help of PyVSvina python script (https://github.com/shuklarohit815/pyVSvina). Before docking, a ‘conf.txt’ file was generated to define the grid box and other parameters. A 3D grid box with the size of 126 Å × 126 Å × 70 Å centered at the coordinates *X* = 192.12, *Y* = 180.89, and *Z* = 269.593 (with a total of 50 genetic runs) was calculated to describe the static conformation of the binding site with default exhaustiveness value 8. Fifteen mutations of RBD omicron variant (B.1.19), G339D, S371L, S373P, S375F, N440K, G446S, T478K, G496S, Q498R, K417N, S477N, E484A, Q493R, N501Y, and Y505H, were defined as the pocket site residues, while other parameters were set as defaults during docking. These key residues were considered for pocket site residues based on different factors including their impact on viral behavior, prevalence in emerging variants, and their clinical significance. Two compounds, propranolol (Thaler et al., 2023) and isoflurane (Landoni et al., 2022) were selected as the positive controls. After screening, the top-ranked molecules were selected based on their binding affinity, number of hydrogen bonds, molecular weight, binding pose, etc. Two macromolecular visualizers, PyMOL v. 2.5.2 (DeLano, 2002) and Discovery studio, were utilized to render and annotate the molecular interactions in 3D and 2D modes, respectively.

### 2.4. Drug-likeness properties

The consensus algorithms of SwissADME (Daina et al., 2017) (http://www.swissadme.ch/) and ADMETlab 2.0 (Xiong et al., 2021)(https://admetmesh.scbdd.com/) were utilized to calculate the drug-likeness properties of the top five screened flavonoid compounds. A set of drug properties including molecular weight; number of hydrogen bond donors and acceptors; rotatable bonds; molar refractivity; bioavailability score; topological polar surface (TPSA); synthetic accessibility; and solubility were calculated for the top-ranked molecules based on the Lipinski’s rule of five (Lipinski, 2004) and Veber’s rule (Veber et al., 2002). The canonical simplified molecular-input line-entry system (SMILES) of top-ranked molecules was utilized in the entry system.

### 2.5. Toxicity analysis

The top-ranked compounds were further subjected to toxicity analysis using ProTox-II (https://tox-new.charite.de/protox_II/), a well-known server for toxicity prediction of small molecules (Banerjee et al., 2018). Generally, this automated webserver calculates the toxicity of individual chemicals based on a set of values such as lethal dose (LD50), toxicity class, average similarity, accuracy prediction, and toxicity against several targets.

### 2.6. Molecular dynamics simulations (MDS)

To evaluate the stability of docking complexes on the atomic level, classical MD simulations were conducted for the top docking complexes of tomentin A, tomentin C, hyperoside, catechin gallate, and corylifol A with RBD of omicron. A GROMOS9643a1 force field, available in the GROMACS 2022 package (Abraham & Gready, 2011; Kutzner et al., 2019) installed on the Linux based workstation, was employed to run the MD simulation on 200 nanoseconds (ns). Topology files of the selected flavonoids were prepared using the automated PRODRG server (https://www.sites.google.com/site/vanaaltenlab/prodrg) (Schüttelkopf & van Aalten, 2004). The docking complexes were contained in a dodecahedron box and solvated with a TIP3P (Transferable Intermolecular Potential with 3 Points) water model as previously described by Jee et al., (2018) and (Kumar et al., 2021). Counter Na + ions were added to neutralize the whole system. The prepared system was minimized with 50,000 iteration steps and cut-off up to 1,000 kjmol^−1^ using the steepest descent algorithm to reduce the clashes during MD simulations. The particle mesh Ewald (PME) truncation algorithm (Abraham & Gready, 2011) was employed to calculate the long-range electrostatic interactions. Equilibration was established with the constant number of particles, volume, and temperature (NVT), and constant number of particles, pressure, and temperature (NPT) with a 500-ps timestep and ensemble at 300 K, respectively. Before the production run, different thermodynamics properties including pressure, density, potential energy, and temperature of the systems were monitored to assess the adequate equilibration. LINCS algorithm was applied to constrain the covalent bonds involving hydrogen atoms (Hess, 2007; Hess et al., 1997). Finally, unrestrained 200 ns production simulations were performed with each step of 2 fs. For this study, we followed the protocol previously described by Gajula et al., (2016).

#### 2.6.1. MDS trajectory analysis

Different factors of MDS such as root mean square deviations (RMSD), root mean square fluctuations (RMSF), number of hydrogen bonds, the radius of gyration (Rg), and solvent accessible surface area (SASA) were studied by plotting histograms after completion of MD simulations on 200 ns. These factors were calculated by using a number of modules available in the GROMACS package.

### 2.7. Estimation of binding free energy

The molecular mechanics/Poisson–Boltzmann surface area (MM/PBSA) method based binding free energy of the top docking complexes was calculated using the g_mmpbsa module developed by Kumari et al. (2014). The g_mmpsa script is integrated with the GROMACS package, accelerating the MD simulations with accurate estimation of binding free energy calculations (Aldeghi et al., 2017). Binding energy, van der Waal energy, electrical energy, polar solvation energy, and SASA were calculated as major components of energy by utilizing the 200 ns long simulation trajectory of the top five flavonoids.

Binding free energy of the top docking complexes were calculated using the following equation:

$$\Delta G_{\text{MMPBSA}}={〈G_{\text{complex}}-G_{\text{protein}}-G_{\text{ligand}}〉}_{\text{complex}}$$

where G_protein_ and G_ligand_ reflect the total free energies of the isolated receptor and ligand molecule in the solvent, respectively, while G_complex_ denotes the total free energy of the protein ligand complex.

### 2.8. Essential dynamics of RBD-complex systems

Essential dynamics (ED) or principal component analysis (PCA), a powerful statistical dimensionality reduction technique, was employed to disseminate the dominant global motions occupied by the structural ensembles of the RBD-complex systems using the last 100 ns MD trajectories employing *gmx covar* and *gmx anaeig* toolkits (Amadei et al., 1993). PCA is often useful to filter the global collective motions occupied by the conformational ensembles from local motions. In this study, we took only the main-chain atoms of each system to generate the covariance matrix plot and, upon diagonalization, a set of eigenvectors and their corresponding eigenvalues were generated, which described the collective modes of fluctuations of biological macromolecules. The eigenvectors corresponding to the largest eigenvalues are called ‘principal components’ or PCs, as they represent the largest-amplitude collective motions.

## 3. Results and discussion

### 3.1. Screening of top interacted natural flavonoid compounds

**Figure 2.**
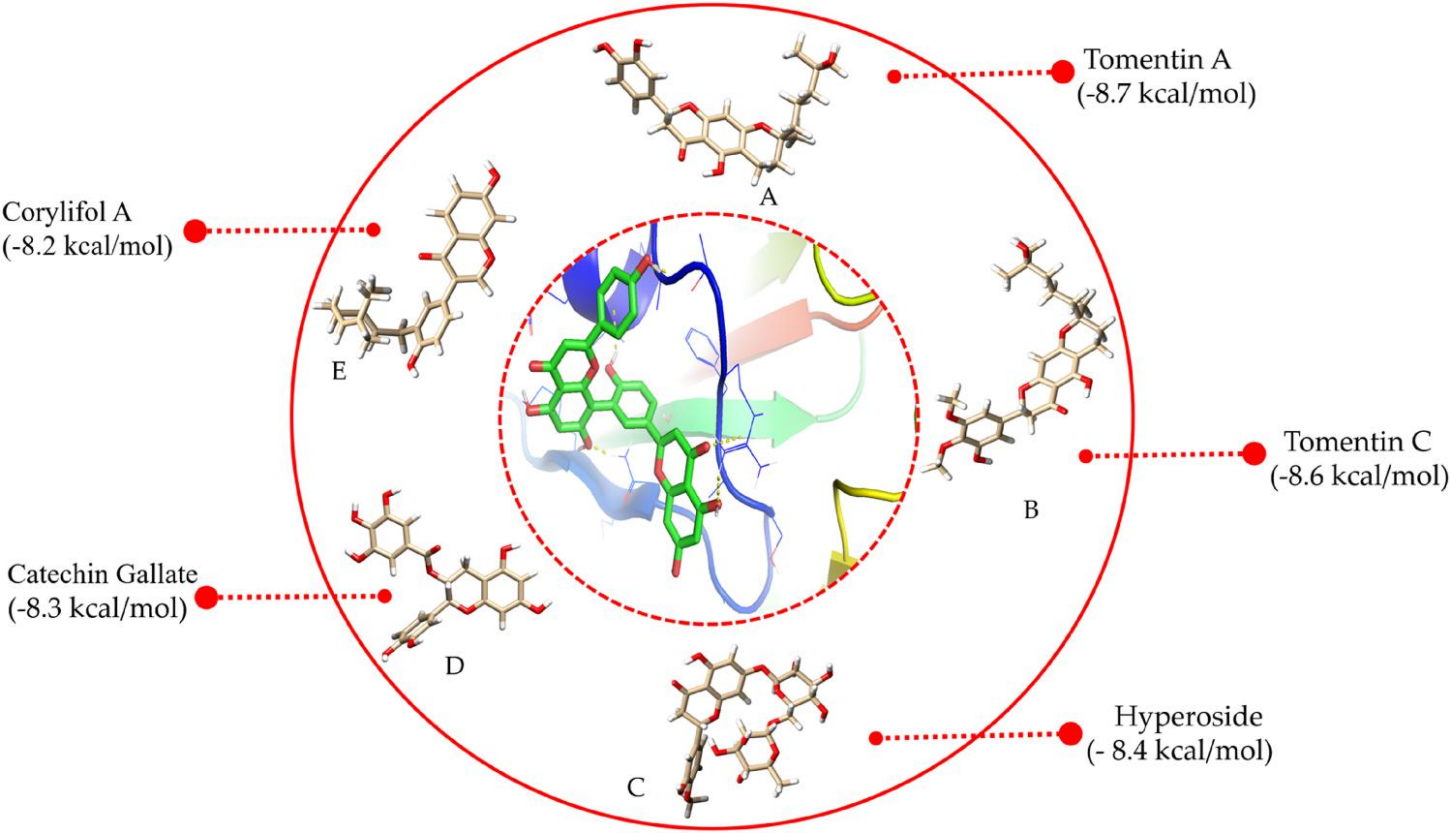
3D representation of the five top-ranked molecules along with their corresponding binding affinity scores calculated as an output of virtual screening. Tomentin A; (B) Tomentin C; (C) Hyperoside; (D) Catechin gallate; and (E) Corylifol A.

**Figure 3.**
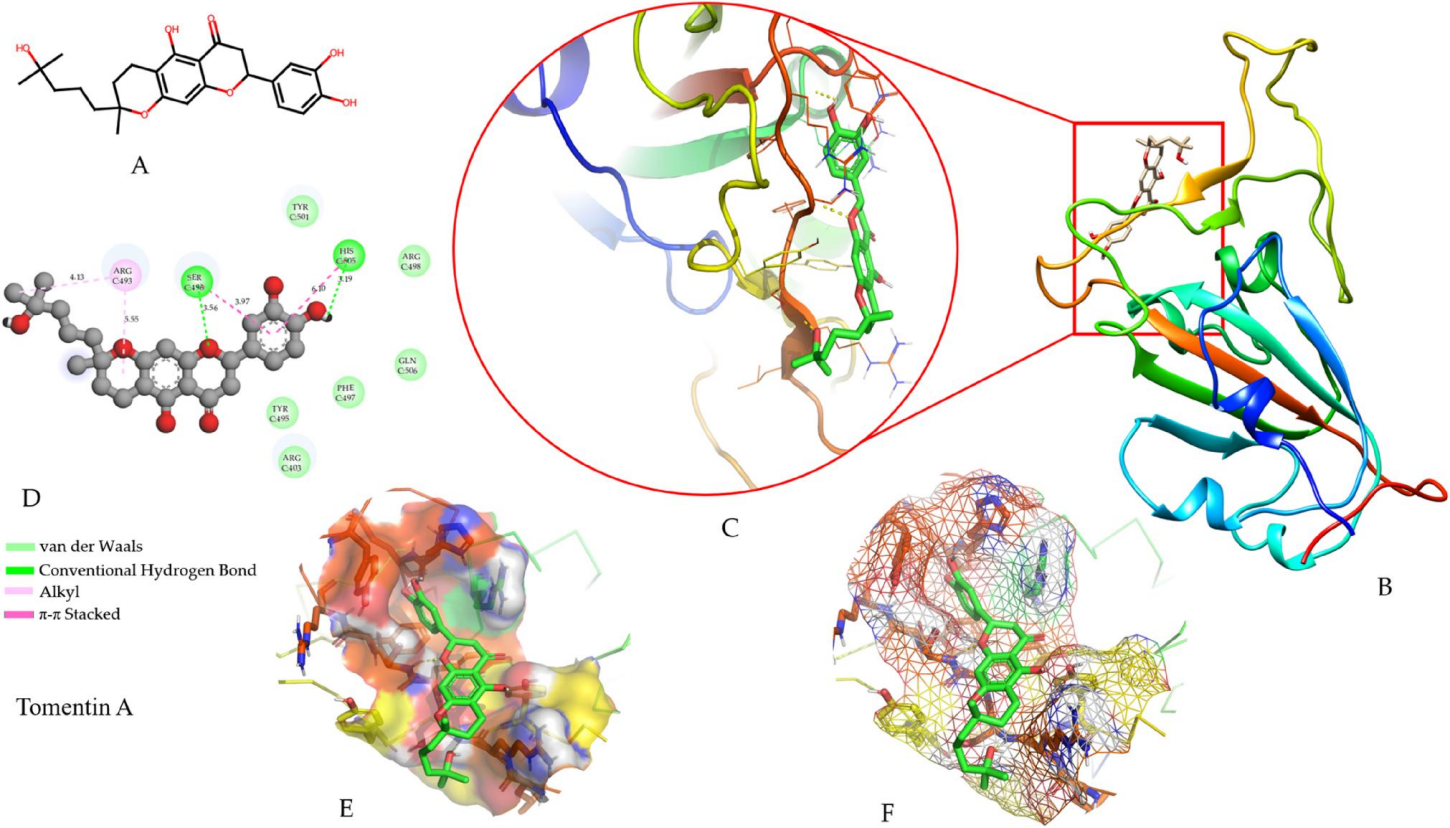
Representation of molecular interactions between tomentin a and omicron RBD. (A) 2D structure of tomentin a derived from the PubChem. (B) Docking complex of tomentin A with 3D structure model of omicron RBD visualized using UCSF-Chimera. (C) Close view of the docking complex to represent the interaction pattern between tomentin A and RBD; yellow dotted lines show the hydrogen bonds; ligand tomentin A denoted with green sticks, and RBD residues shown as atom-type color sticks. (D) Discovery Studio rendered 2D plot of docking complex with different interaction representations including conventional hydrogen bond, van der Waals, alkyl, and π-π stacked. (E) Solid transparent representation. (F) Mesh surface view of tomentin A and RBD using PyMOL.

Over the past decades, the molecular docking based virtual screening approach has expanded dramatically in the field of computer-aided drug design (CADD) to screen potential drug candidates from the libraries of small molecules against a plethora of contagious diseases. There is still a need for an effective inhibitor for SARS-CoV-2; therefore, screening of potential therapeutics is required on an urgent basis to overcome this necessity. In the present study, we examined the inhibition potential of fruit and vegetable-derived natural flavonoids against RBD of omicron using an extensive molecular modelling approach. Forty natural flavonoids were docked with the mutated residues of RBD. The virtual screening results revealed that out of the 40 natural flavonoids analyzed, five natural compounds, tomentin A, tomentin C, hyperoside, catechin gallate, and corylifol A, were found to have higher negative binding energies of −8.7, −8.6, −8.4, −8.3, and −8.2 kcal/mol, respectively (Figure 2 and Supplementary Table 1). These top-ranked molecules were also found to have higher negative binding energies than the positive control compounds propranolol and isoflurane, whose binding energies were determined to be −6.1 and −4.4 kcal/mol, respectively. The screened top-ranked molecules were selected based on several parameters including binding energy score (≥ 8.0 kcal/mol^−1^), number of hydrogen bonds with mutated residues (≥ 2), and molecular weight (≤ 500 g/mol) as a result of virtual screening. 2D and 3D plots of interaction patterns were generated for these top-ranked compounds. After exploring the interaction patterns, we determined that tomentin A formed two hydrogen bonds with Ser496 (3.56 Å) and His505 (3.19 Å). Residue Arg493 (4.13, 5.55 Å) formed an alkyl bond with tomentin A. Two residues, Ser496 (3.97 Å) and His505 (6.10 Å), demonstrated π-π stacked bonds. Tomentin A compound was found to have six van der Waals (vdW) bonds with Arg403, Tyr495, Phe497, Arg498, Tyr501, and Gln506 residues (Figure 3).

In the tomentin C molecule, two residues, Ser494 (3.32 Å) and His505 (4.09 Å), exhibited hydrogen bond interactions. The residues Arg493 (4.42, 5.72 Å), His505 (4.42 Å), and Arg403 (6.31 Å) showed carbon hydrogen (C-H), π-sigma, and π-cation interactions, respectively. Two residues, Tyr501 (4.16 Å) and His505 (4.09 Å), formed π alkyl bonds. Six residues, Tyr453, Leu455, Leu492, Tyr495, Ser496, and Phe497, manifested (vdW) interactions (Supplementary Figure 1(A,E)). As shown in Supplementary Figure 1(B,F), Arg403 (5.82, 6.12 Å) and Ser496 (3.44, 4.47 Å) residues formed hydrogen bonds with the hyperoside compound. Arg493 (3.98 Å) and Ser496 (4.14 Å) residues showed C-H and π-donor hydrogen bonds, respectively. Arg403 (5.92 Å) residue manifested a single π-cation bond. Eight residues, Tyr449, Tyr453, Ser494, Leu455, Tyr495, Phe497, Arg498, and Tyr501, demonstrated vdW interactions.

In the case of catechin gallate, four residues, namely, Arg403 (4.17 Å), Ser494 (4.49 Å), Ser496 (3.85 Å), and His505 (4.24 Å), manifested hydrogen bonds. Tyr501 (5.66 Å) was found to interact through a π-π T-shaped bond. Four residues, Tyr453, Tyr495, Phe497, and Arg498, interacted with the catechin gallate *via* vdW (Supplementary Figure 1(C,G)). As evident from Supplementary Figure 1(C,G), Ser496 (4.09 Å) formed a single hydrogen bond with the corylifol A molecule. Tyr501 residue was found to interact through a π-donor hydrogen bond and π-π T-shaped interaction with distances of 3.33 and 5.53 Å, respectively. Residues Arg493 (4.09 Å), Arg403 (6.13 Å), and His505 (5.53 Å) showed alkyl, π-cation, and π-π stacked interactions, respectively. Seven residues (Tyr449, Tyr453, Ser494, Tyr495, Phe497, Arg498, and Gln506) were involved in vdW interactions, stabilizing the RBD-corylifol A complex in a significant manner (Supplementary Figure 1(D,H)). Table 1 represents the 2D structures of the control molecules and top five molecules along with their common names, chemical IDs, molecular formulae, binding energy scores, and molecular interactions information. Interestingly, all five shortlisted compounds (i.e. tomentin A, tomentin C, hyperoside, catechin gallate, and corylifol A) were found to have a common hydrogen bond/or C-H bond interaction with Ser496 residue. These top-ranked molecules also shared interaction patterns with the non-mutated residues; this additional binding indicates strengthened bonds, thus showing a tight fit to RBD. The functional hydroxyl group (—OH) of flavonoids, attached to a carbon atom in a benzene ring, was found dominant in top-ranked compounds to have more hydrogen bond interaction against receptor RBD during interactions profiling. The overall docking-based interaction analysis revealed that all top five compounds demonstrated notable interaction patterns with RBD of omicron. The natural flavonoids (tomentin A, tomentin C, hyperoside, catechin gallate, and corylifol A) reported in the present study may support previous findings on the inhibition potential of phytochemicals against RBD of omicron.

**Table 1.** List of the control compounds and top five natural compounds.

Name	PubChem ID	Molecular Formula	Binding Energy (kcal/mol)	2D Structure	Molecular Interactions
Propranolol (Control 1)	4946	C_16_H_21_NO_2_	−6.1	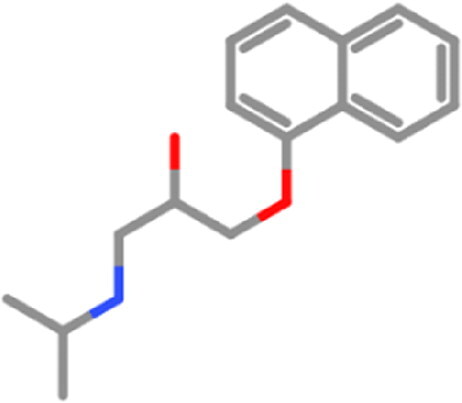	**Hydrogen Bond:** Arg403 (4.57 Å), Ser494 (4.60, 4.69 Å) **Alkyl:** Arg493 (4.18 Å) **π**-**π Stacked/T Shaped: Tyr501** (6.60 Å), **His505** (4.74, 5.55 Å) **van der Waals:** Tyr453, Tyr495, **Ser496**, Phe497
Isoflurane (Control 2)	3763	C_3_H_2_ClF_5_O	−4.4	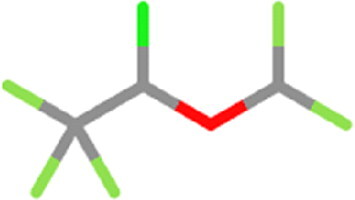	**Carbon Hydrogen:** Arg403 (4.02 Å) **π Alkyl:** Tyr495 (4.49 Å), **Tyr501** (5.04 Å), **His505** (4.46 Å) **Alkyl:** Arg403 (5.00 Å) **van der Waals:** Ser496, Phe497, **Arg498**
Tomentin A	71659627	C_25_H_30_O_7_	−8.7	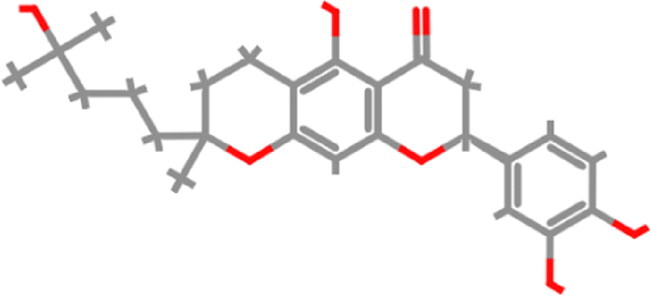	**Hydrogen Bond**: **Ser496** (3.56 Å), **His505** (3.19 Å) **Alkyl: Arg493** (4.13, 5.55 Å) **π**-**π Stacked: Ser496** (3.97 Å), **His505** (6.10 Å) **van der Waals:** Arg403, Tyr495, Phe497, **Arg498**, **Tyr501**, Gln506
Tomentin C	71659765	C_27_H_34_O_8_	−8.6	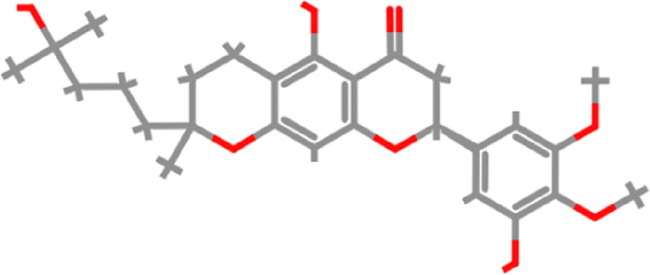	**Hydrogen Bond:** Ser494 (3.32 Å), **His505** (4.09 Å) **Carbon Hydrogen: Ser496** (4.90 Å) **Alkyl: Arg493** (4.42, 5.72 Å) **π Alkyl: Tyr501** (4.16 Å), **His505** (4.09 Å) **π Sigma**: **His505** (4.42 Å) **π Cation:** Arg403 (6.31 Å) **van der Waals:** Tyr453, Leu455, Leu492, Tyr495, **Ser496**, Phe497
Hyperoside	5281643	C_21_H_20_O_12_	−8.4	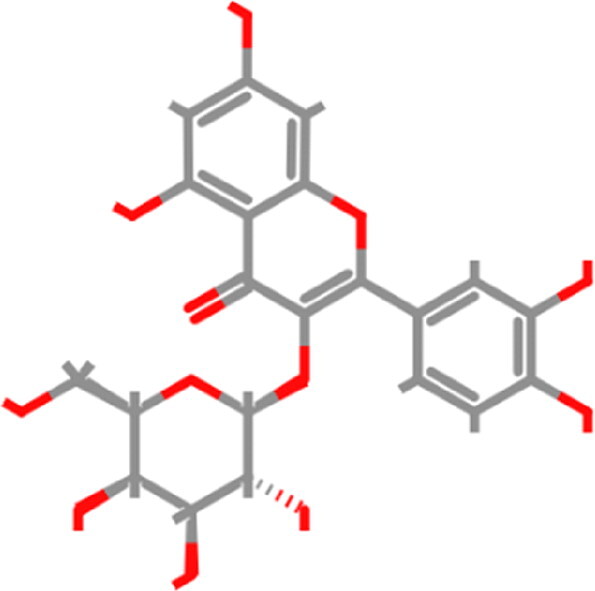	**Hydrogen Bond**: Arg403 (5.82, 6.12 Å), **Ser496** (3.44, 4.47 Å) **Carbon Hydrogen Bond**: **Arg493** (3.98 Å) **π-Donor Hydrogen Bond**: **Ser496** (4.14 Å) **π-Cation**: Arg403 (5.92 Å) **van der Waals**: Tyr449, Tyr453, Ser494, Leu455, Tyr495, Phe497, **Arg498**, **Tyr501**
Catechin gallate	6419835	C_22_H_18_O_10_	−8.3	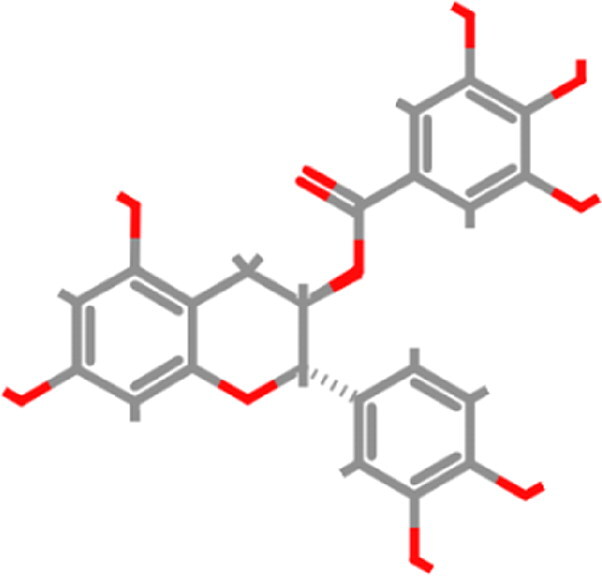	**Hydrogen Bond**: Arg403 (4.17 Å), Ser494 (4.49 Å), **Ser496** (3.85 Å), **His505** (4.24 Å) **π**-**π T-Shaped: Tyr501** (5.66 Å) **van der Waals:** Tyr453, Tyr495, Phe497, **Arg498**
Corylifol A	25056407	C_25_H_26_O_4_	−8.2	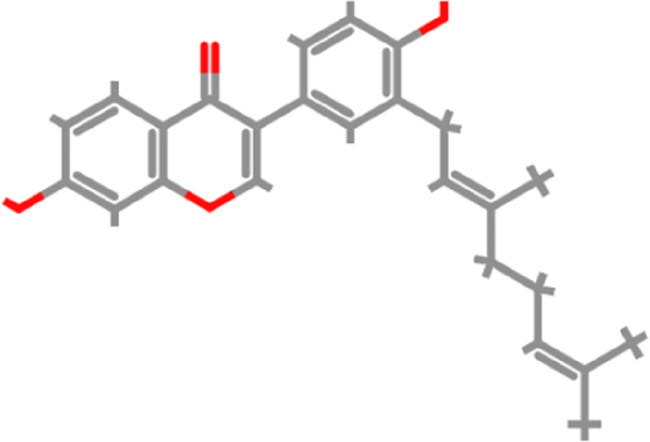	**Hydrogen Bond**: **Ser496** (4.09 Å) **π Donor Hydrogen Bond: Tyr501** (3.33 Å) **Alkyl: Arg493** (4.09 Å) **π Cation:** Arg403 (6.13 Å) **π**-**π T-Shaped: Tyr501** (6.50 Å) **π**-**π Stacked: His505** (5.53 Å) **van der Waals:** Tyr449, Tyr453, Ser494, Tyr495, Phe497, **Arg498**, Gln506

These top five compounds were shortlisted based on the molecular weight, binding energy score, and number of hydrogen bonds with mutated residues as a result of virtual screening.

### 3.2. Drug-likeness properties of the top-ranked compounds

Drug likeness properties of the top-ranked molecules (tomentin A, tomentin C, hyperoside, catechin gallate, and corylifol A) were evaluated based on Lipinski’s rule of five. This method is important to determine if a molecule is likely to have physiochemical properties to be orally bioavailable (Lipinski, 2004). Per Lipinski’s rule of five, an active chemical is considered as drug-like if it possesses the following properties: molecular weight (mw) ≤ 500; number of H-bond acceptors ≤10; number of H-bond donors ≤5; LogP ≤5; and molar refractivity from 40 to 130 (Lipinski, 2004). Calculated ADME properties of the top-ranked molecules in the present study are shown in Supplementary Table 2. Tomentin A, tomentin C, hyperoside, catechin gallate, and corylifol A have the following mw, respectively: 442.50, 486.55, 464.38, 442.37, and 390.47 g/mol; these five natural flavonoids have a mw ≤ 500, so they can smoothly be transported, diffused, and absorbed. Out of the top five compounds, three molecules namely, tomentin A, tomentin C, and corylifol A, had numbers of H-bond acceptors ≤10, and numbers of H-bond donors ≤5, while two other molecules, hyperoside and catechin gallate, did not meet the conditions of H-bond acceptors and donors. The LogP values of tomentin A, tomentin C, hyperoside, catechin gallate, and corylifol A molecules were found to be 3.59, 3.97, −0.25, 1.25, and 5.15, respectively, which meets the essential conditions of drug likeness. The tomentin A, tomentin C, hyperoside, catechin gallate, and corylifol A have molar refractivity scores of 120.61, 131.57, 110.16, 110.04, and 119.25, respectively; these molecules also present the values of synthetic accessibility: 4.56, 4.88, 5.32, 4.16, and 3.86, respectively. Evaluation of molar refractivity (range 40-130) and synthetic accessibility (range ≤6) revealed that all five flavonoids meet the criteria and can be synthesized easily and thus considered as drug-like molecules. The drug likeness calculations indicated that tomentin A, tomentin C, hyperoside, catechin gallate, and corylifol A present the following values of the TPSA: 116.45, 114.68, 210.51, 177.14, and 70.67 Å2, respectively, which are the conformation TPSA properties of natural compounds previously reported against SARS-CoV-2 (Kumar et al., 2021; Kumar et al., 2021). With the exception of the hyperoside and catechin gallate compounds, these natural flavonoids also validate Veber’s rule. ADMET2.0 based systematic evaluation of different physicochemical properties for all five top-ranked molecules are shown in Supplementary Figure 2.

### 3.3. Toxicity analysis

Preliminary toxicity evaluation of small compounds using *in-silico* models is important to reduce the time and costs of screening potential drug candidates and to design animal testing protocols (Raies & Bajic, 2016). Toxicity of the top-ranked compounds was predicted using automated ProTox-II with the estimation of different key parameters including hepatotoxicity, carcinogenicity, mutagenicity, immunotoxicity, cellular toxicity, toxicological pathways (Tox21-Nuclear receptor signalling and Tox21-stress response pathways), and acute oral toxicity (LD_50_). Calculated toxicity profiles of tomentin A, tomentin C, hyperoside, catechin gallate, and corylifol A are provided in Supplementary Table 3. All top five compounds were predicted as non-carcinogenic and non-cytotoxic in nature and found to be safe in most of the toxicity parameters. Three compounds, tomentin A, tomentin C, and catechin gallate, belong to class 4 with LD_50_ values ranging from 1000 to 2000 mg/kg, making these compounds harmful for oral delivery. Two compounds, hyperoside and corylifol A, belong to class 5 with LD_50_ values of 5000 and 2500 mg/kg, respectively. Compounds belonging to class 5 may be harmful if swallowed.

### 3.4. All five shortlisted flavonoids showed stability with RBD on 200 ns

To understand the binding mode patterns, dynamic motion, and stability of the docking complexes, the top five screened flavonoids, tomentin A, tomentin C, hyperoside, catechin gallate, and corylifol A, that passed the drug-likeness properties and showed potential binding energy were considered for MD simulations analysis.

#### 3.4.1. RMSD

**Figure 4.**
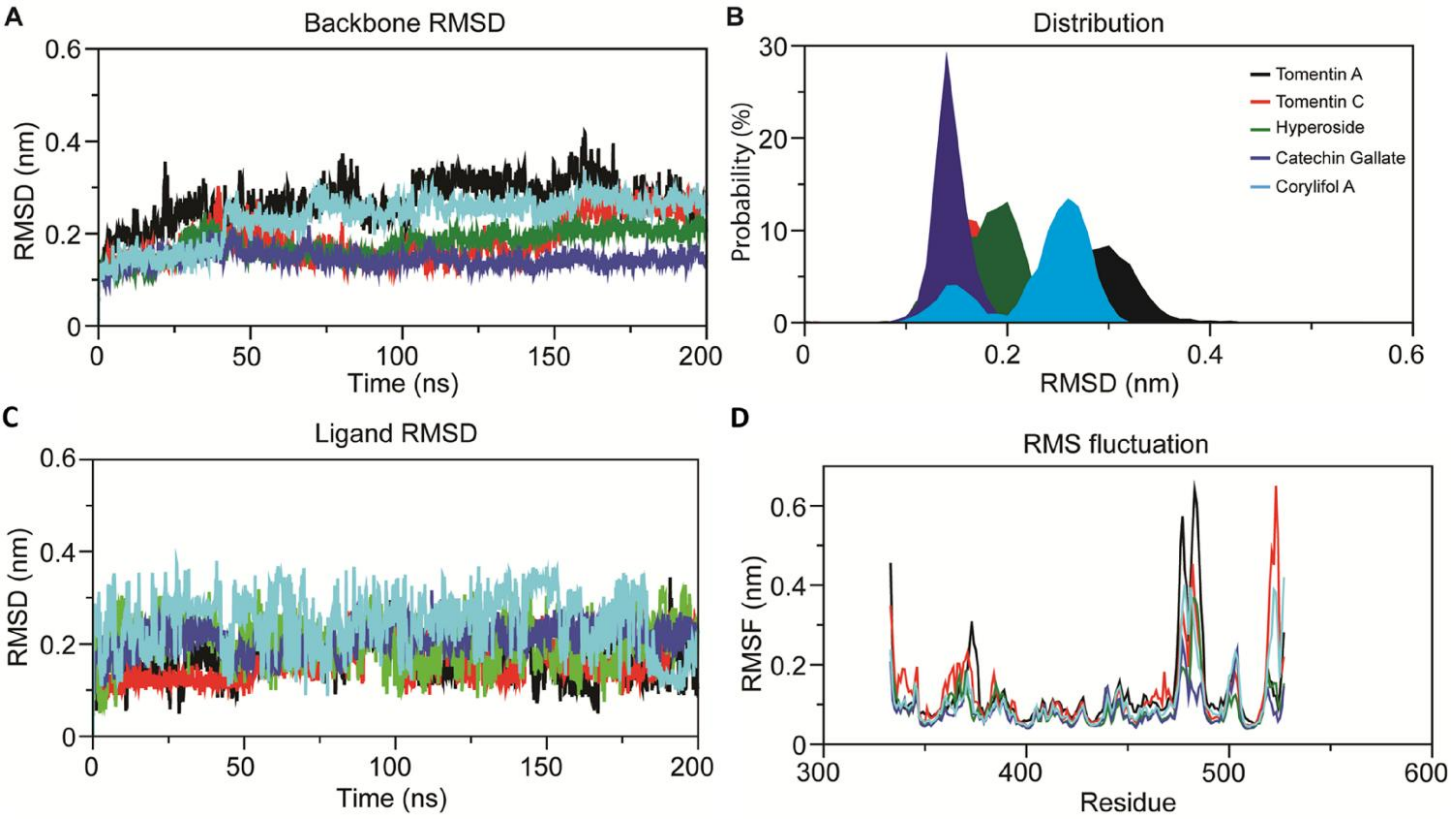
Intrinsic dynamics stabilities of the top-ranked natural flavonoids (tomentin A, tomentin C, hyperoside, catechin gallate, and corylifol A) in complex with omicron RBD during all-atoms MD simulations on 200 ns. Colors represented on the bottom panel are followed in the below images as well. (A) Calculated backbone RMSD plot of docking complexes. (B) Probability distribution plot of complexes. (C) Ligand RMSD plot of docking complexes. (D) RMS fluctuation plot of docking complexes [RBD-tomentin A (black), RBD-tomentin C (red), RBD-hyperoside (green), RBD-catechin gallate (purple), and RBD-corylifol A (blue)]. the RMSD and RMSF plots of protein-ligand complexes were estimated using gRMS and gRMSF tools, respectively.

RMSD has been defined as one of the quantitative methods to determine the stability of protein-ligand complexes and calculate conformational stability in the protein backbone during MDS within the time frame (Sargsyan et al., 2017). RMSD values were calculated for all five complexes and graphically measured. The calculated backbone RMSD value ranged from ∼0.1 to ∼0.4 nm for all five system trajectories. As evident from Figure 4, four ligands, specifically tomentin C (red), hyperoside (green), catechin gallate (purple), and corylifol A (blue), exhibited a constant range of stability from initiation point to ∼40 ns. The complexes with tomentin A (black) and corylifol A (blue) depicted higher simulation trajectories after ∼50 ns than the complexes with tomentin C (red), hyperoside (green), and catechin gallate (purple). Tomentin C (red) displayed two fluctuations throughout the MDS on 200 ns; the first significant stable conformation was recorded between 50 to 120 ns, while the second was noted between 160 to 200 ns. The catechin gallate (purple) showed a straight line without any significant fluctuations during simulations and reflected minimum conformational changes as compared to the four other compounds. The calculated ligand RMSD plot also demonstrated that the small conformational changes and RMSD values ranged from 0.14 to 0.27 nm for all five complexes. The average RMSD values for the RBD complexes with tomentin A, tomentin C, hyperoside, catechin gallate, and corylifol A were 0.27, 0.18, 0.17, 0.14, and 0.23 nm, respectively (Supplementary Table 4). As depicted in Figure 4(C), corylifol A (blue) showed the highest simulation trajectory throughout the MDS as compared to the other four molecules. Four molecules, tomentin A (black), tomentin C (red), hyperoside (green), and catechin gallate (purple), demonstrated a constant range of stability between 5 ns to 50 ns and 150 to 180 ns and significantly contributed to the stability of the complexes. A calculated probability distribution plot revealed that catechin gallate (purple) exhibited the highest peak of stability, between 0.1 to 0.2 nm, compared to the other molecules (Figure 4(B)**)**. Information obtained from the backbone and ligand RMSD plots illustrated the stability of simulation trajectories with minimum conformational changes.

#### 3.4.2. RMSF

RMSF is the most commonly used method to measure individual residue flexibility of a system during MDS (Farmer et al., 2017). The average RMSF values of the RBD-tomentin A, RBD-tomentin C, RBD-hyperoside, RBD-catechin gallate, and RBD-corylifol A docking complexes were 0.13, 0.12, 0.09, 0.08, and 0.10 nm, respectively (Supplementary Table 4**)**. A low value indicates the more stable structure segment in the docking complexes (Gajula et al., 2016). As shown in Figure 4(D), very high fluctuations were noted in RBD-tomentin A and RBD-tomentin C complexes as compared to the other three docking complexes. At the end point, a calculated RMSF plot showed remarkable fluctuations, which may be due to loops in the protein structure. In a previous report, (Farouk et al., 2021) also reported the same type of fluctuation patterns in RBD for the 365–385 residues, 415–435 residues, and the loop region residues (477–485). Consistent with the molecular docking results, RMSF plot calculations indicated that the residues distributed across the pocket site of RBD significantly interact with tomentin A, tomentin C, hyperoside, catechin gallate, and corylifol A.

#### 3.4.3. Hydrogen bond interaction analysis

**Figure 5.**
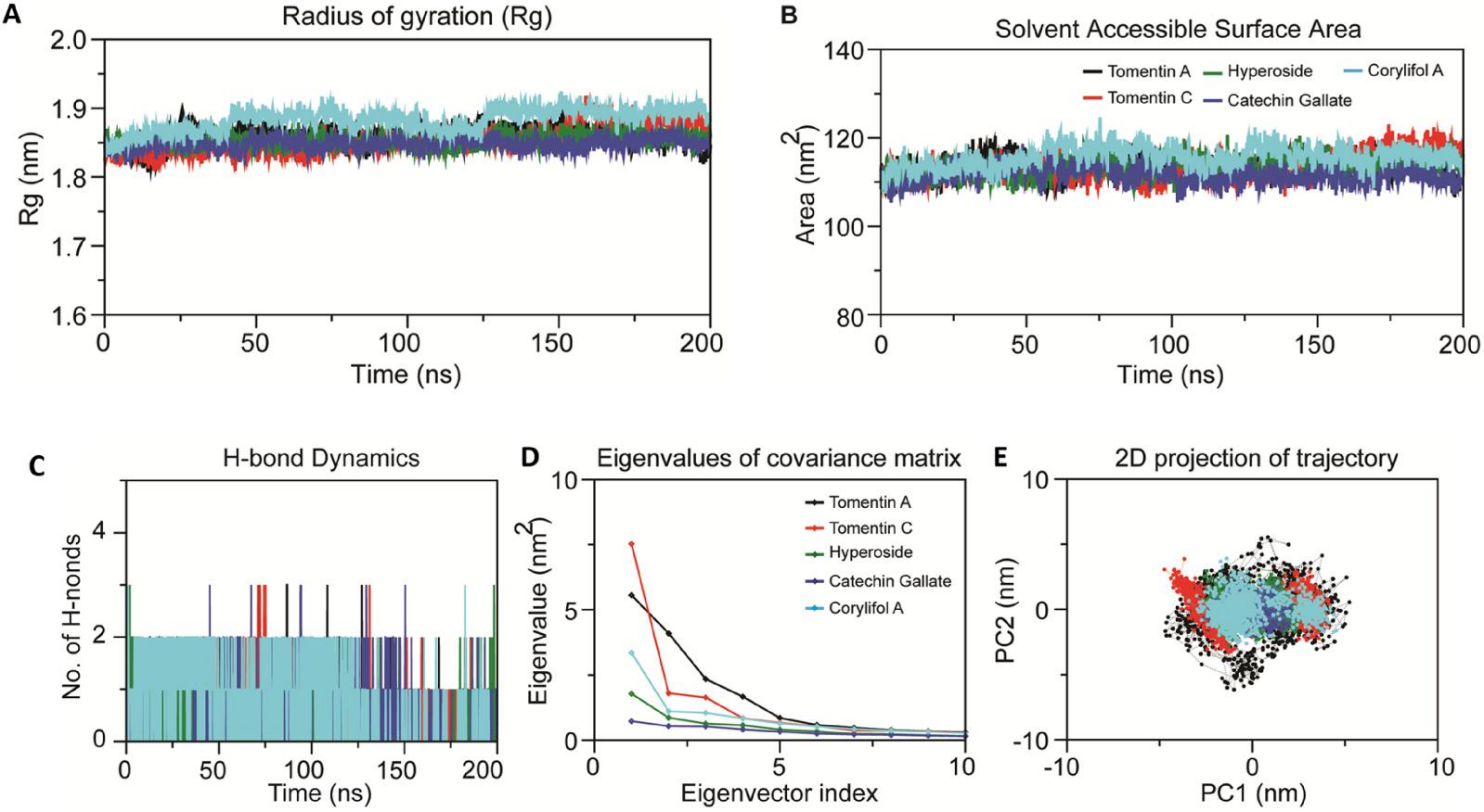
Dynamics of Rg, SASA, intermolecular hydrogen bonds, and PCA analysis of the omicron RBD complexes with tomentin A, tomentin C, hyperoside, catechin gallate, and corylifol a during all-atoms MD simulations on 200 ns. (A) Rg of omicron RBD with compounds over the time scale of 200 ns. (B) Calculated SASA plot of docking complexes. (C) Intermolecular H-bond dynamics plot of docking omicron RBD-flavonoids complexes during 200 ns. Rg plot was calculated using gyrate, while the SASA was generated using gmxsasa module. Hydrogen bonds were estimated using g h bond tool. (D) Eigenvalues vs. eigenvectors plot estimated with the main-chain atoms covariance matrix constructed from the last 100 ns of the MD trajectories for RBD-complex systems. (E) 2D projection plot of trajectory motion of the omicron RBD-complex systems [RBD-tomentin A (black), RBD-tomentin C (red), RBD-hyperoside (green), RBD-catechin gallate (purple), and RBD-corylifol A (blue)] in phase space along with the PC1 and PC2.

Measurement of hydrogen bond interactions is one of the crucial steps toward understanding and annotating the molecular interaction patterns of protein-ligand complexes using MDS methods (Chikalov et al., 2011; Dutt et al., 2023). In this present study, we calculated a number of hydrogen bonds from MDS trajectories to determine the stability between RBD and screened the top-ranked flavonoids and plotted them in Figure 5(C). It was observed from the calculated plot that all five ligand molecules (tomentin A, tomentin C, hyperoside, catechin gallate, and corylifol A) formed an average of two hydrogen bonds up to 150 ns. After 150 ns, all five compounds lost one hydrogen bond up to 180 ns; thereafter, only hyperoside and corylifol A gained hydrogen bonds that maintained until the end of the simulation. Hydrogen bond interaction analysis revealed that all five molecules exhibited a strong pattern of interaction with RBD and are the conformation of docking results. The overall hydrogen bond analysis indicated that all five molecules significantly participated in the stabilization of the system by forming hydrogen bonds with the RBD throughout the simulation.

#### 3.4.4. Rg and SASA

MD trajectories of complexes RBD-tomentin A, RBD-tomentin C, RBD-hyperoside, RBD-catechin gallate, and RBD-corylifol A were calculated for Rg and SASA analysis. As shown in Figure 5(A), on average, Rg values of the docking complexes ranged between ∼1.8 and ∼1.9 nm throughout the simulation on 200 ns. The average Rg value of RBD-tomentin A, RBD-tomentin C, and RBD-hyperoside complexes was 1.85 nm, while RBD-catechin gallate and RBD-corylifol A complexes showed average values of 1.84, and 1.88 nm, respectively. RBD-catechin gallate (purple) complex showed the lowest Rg value as compared to the other shortlisted complexes. The Rg of RBD-corylifol A (blue) showed a set of small fluctuations and primarily decreased between ∼70 to ∼125 ns on ∼1.8 nm, but the Rg value increased during the ∼130 and 200 ns simulation on ∼1.90 nm. The measured Rg values for these top-ranked molecules are consistent with previous reports which have explored the inhibition potential of phytochemicals as effective inhibitors against RBD of omicron (Hakami, 2022; Lü et al., 2022). In the present study, the relatively consistent Rg values depicted the stability of complexes throughout the MD simulation period.

The rapid and accurate estimation of SASA is useful in measuring receptors exposed to solvents during MDS on a time scale. The calculated SASA plot for the top-ranked flavonoid molecules and RBD is give in Figure 5(B). The evaluated SASA values ranged between 111 and 115 nm^2^. The average SASA values of RBD-tomentin A, RBD-tomentin C, RBD-hyperoside, RBD-catechin gallate, and RBD-corylifol A docking complexes were 113.60, 113.33, 113.55, 111.58, and 115.61 nm^2^, respectively (Supplementary Table 4**)**. As expected, RBD-tomentin A, RBD-tomentin C, and RBD-hyperoside exhibited relatively consistent patterns of SASA values. RBD-catechin gallate (purple) complex showed the lowest SASA value, while RBD-corylifol A (blue) demonstrated the highest value as compared to the other evaluated complexes. Altogether, the measured SASA plot indicates the stability of the complexes throughout the simulation and also exhibits that the binding of tomentin A, tomentin C, hyperoside, catechin gallate, and corylifol A does not affect the folding of the receptors in a significant manner.

### 3.5. Essential dynamics and porcupine analysis

**Figure 6.**
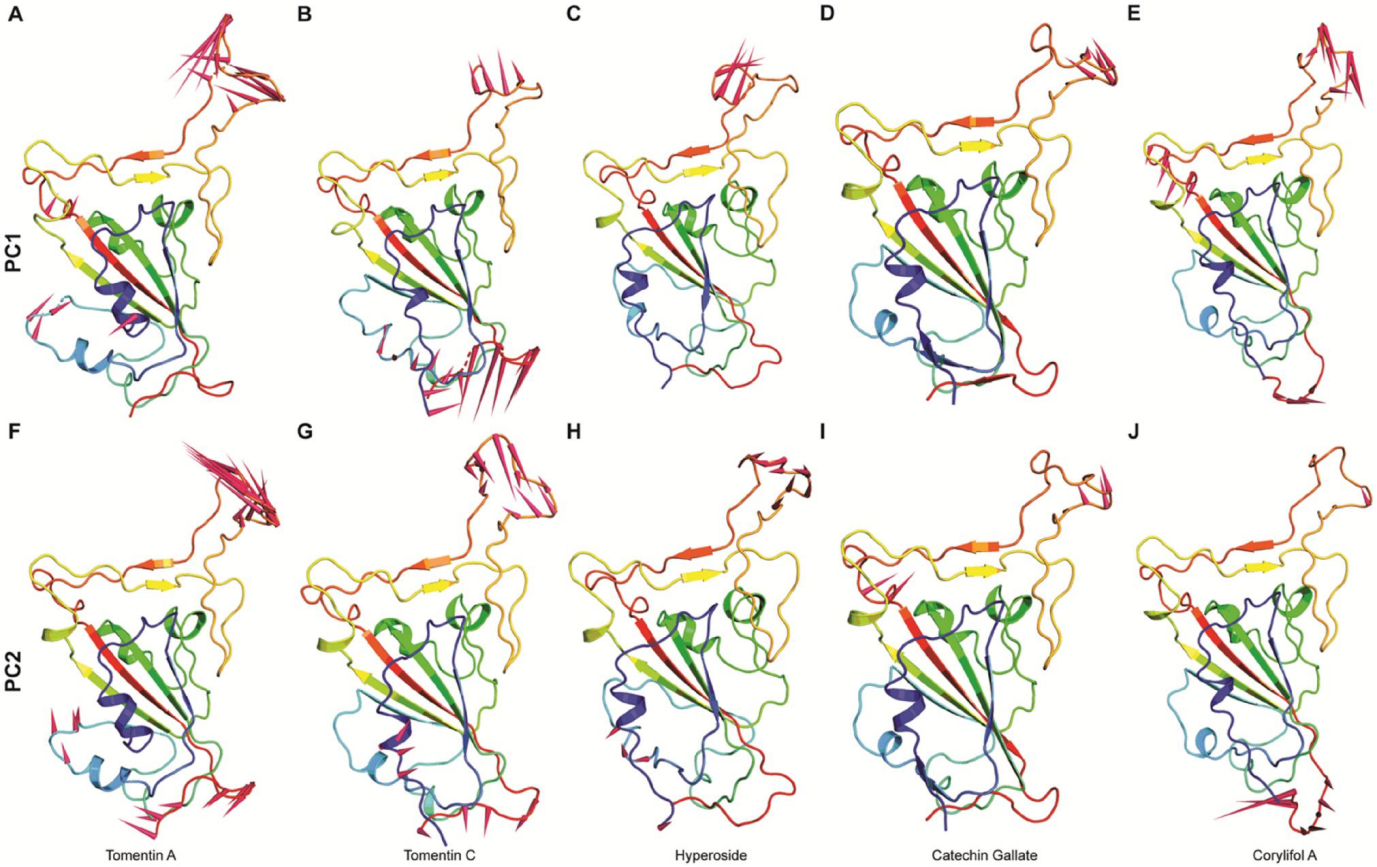
Calculated porcupine plots for the top two PCs. The direction of the arrows represents the magnitude of the motion.

The diagonalization of the covariance matrices of each system yielded a set of eigenvectors and their corresponding atomic fluctuations Figure 5(D). From the eigenvalue plot (shown in Figure 5(D)), it can be detected that the first four eigenvalues are relative to concerted motions rapidly declining in amplitude to reach a stable plateau with more localized fluctuations. The 2D projection plots from PCA also suggest that the first 8 to 10 PC account for more than ∼93% of the total motions observed in the last 100 ns of the MD trajectories for the RBD-complex systems. We then projected the first two EVs (EV1 and EV2) into the phase space where the tomentin A complex occupies a great degree of conformational space followed by tomentin C, while, hyperoside, catechin gallate, and corylifol A complexes displayed the least motion among the five systems studied. Interestingly, the least differential scattering of main-chain atoms observed in this study indicates that minor conformational changes occur in all complexes during MD (Figure 5(E)). To understand the degree of flexibility in each system, we generated porcupine plots for the top two PCs (as illustrated in Figure 6). We observed that the loop connecting the β-strand in the binding interface displayed a high degree of mobility in both PC1 and PC2 of all the systems, signifying the importance of this segment in the molecular recognition process. Furthermore, both terminal ends also displayed outward motion. Among the systems, tomentin A, tomentin C, and corylifol A occupied more conformational space with high trace values (i.e. 21.12 nm^2^, 19.14 nm^2^, 13.37 nm^2^, respectively) as compared to the other two complexes, (i.e. catechin gallate 6.47 nm^2^ and hyperoside 8.52 nm^2^).

### 3.6. Post MD interaction analysis

**Figure 7.**
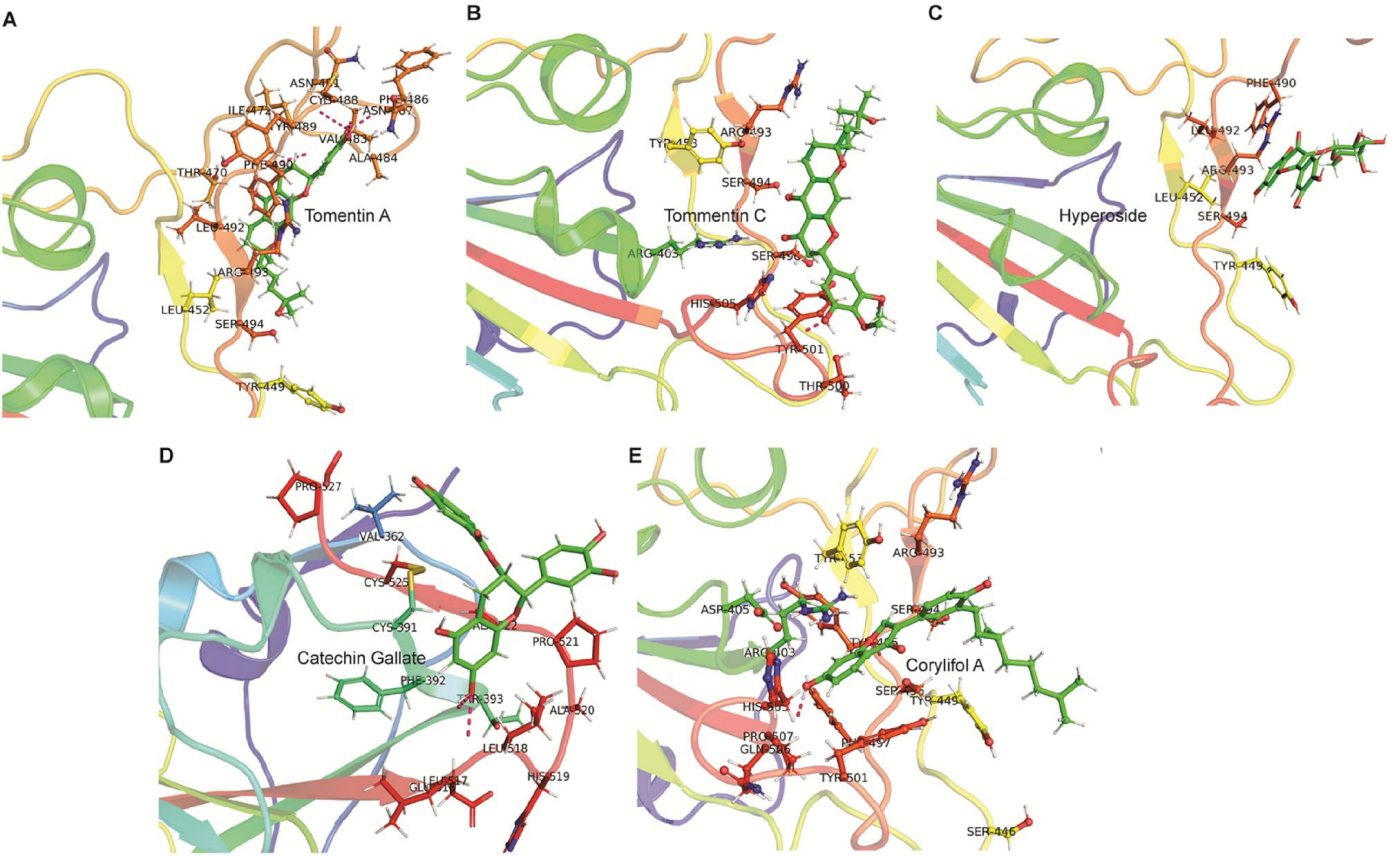
3D representation of post MD simulations interaction patterns between the five top-ranked flavonoids and omicron RBD on 200 ns. Red dotted lines show the hydrogen bond interactions between RBD and natural flavonoids, while, residues of the RBD are denoted by atom-type color sticks. **(A)** RBD-tomentin A; **(B)** RBD-tomentin C; **(C)** RBD-hyperoside; **(D)** RBD-catechin gallate; and **(E)** RBD-corylifol A.

To gain more insight into protein-ligand conformations, post MD simulation interaction analysis was conducted for all five top-ranked molecules in complex with RBD. During interaction analysis, a number of molecular interactions including conventional hydrogen bond, carbon hydrogen bond, π-donor hydrogen bond, π-cation, π-anion, π-lone pair, π-π stacked, π-alkyl, alkyl, π-sigma, π-sulfur, etc. were extensively analyzed for binding patterns with RBD on 200 ns. Supplementary Table 5 presents the detailed molecular interactions between the screened flavonoids (tomentin A, tomentin C, hyperoside, catechin gallate, and corylifol A) and omicron RBD. Two compounds (tomentin A and catechin gallate) showed the highest number of conventional hydrogen bonds with RBD as compared to the other screened molecules. As shown in Figure 7, tomentin A formed hydrogen bonds with Cys488 (2.25 Å), Asn481 (2.68 Å), Val483 (2.15 Å), and Phe486 (1.91 Å). Two residues, Phe490 (5.34 Å) and Ala484 (5.02 Å), manifest π-alkyl interactions. The residues Phe490 (4.21 Å) and Leu452 (5.26 Å) showed π-π stacked and alkyl interactions with tomentin A. In the case of catechin gallate, Thr393 (2.84, 2.91 Å) and Ala522 (1.95 Å) formed the hydrogen bonds. Residues Pro521 (2.55 Å) and Ala522 (2.75 Å) exhibited π-sigma interactions. Cys391 (5.09, 4.66 Å) residues were found to interact with alkyl and π-alkyl interactions. Residues Ala522 (4.21 Å) and Leu518 (4.84 Å) interacted *via* alkyl and π-alkyl interactions. The catechin gallate compound also showed two π-sulfur interactions with Cys391 (4.00 Å) and Cys525 (3.75 Å) residues. Other compounds also showed a number of intermolecular interactions with RBD on 200 ns. Tomentin C manifests one hydrogen bond with Gly502 (1.95 Å), and π-alkyl interacts with Tyr501 (4.89 Å). Three residues, Ser494 (1.92 Å), Arg493 (2.55 Å), and Phe490 (4.83 Å), demonstrated hydrogen bonds, carbon hydrogen bonds, and π-π stacked interactions with hyperoside compound, respectively. The corylifol A molecule formed two hydrogen bonds with Ser496 (1.70 Å) and His505 (1.91 Å) residues. Three compounds, Ser496 (2.59 Å), Arg403 (3.48, 3.66 Å), and Asp405 (4.98 Å), interacted *via* π-donor hydrogen bonds, π-cation, and π-anion, respectively. Corylifol A molecule exhibited π-alkyl interactions with Tyr449 (5.35 Å) and Arg403 (4.78 Å) residues. Two residues, Tyr501 (5.01 Å) and Ser496 (2.99 Å), showed π-π stacked and π-lone pair interactions with corylifol A, respectively. As compared with the docking findings, post MD interaction analysis showed some additional types of interactions, leading to strong molecular interactions with omicron RBD at the atomic level.

### 3.7. Binding free energy analysis

The binding free energy of all top-ranked complexes (RBD-tomentin A, RBD-tomentin C, RBD-hyperoside, RBD-catechin gallate, and RBD-corylifol A) was calculated using the MmPbSaStat.py available in the g_mmpbsa tool. The most popular MM/PBSA approach was applied to calculate the binding free energy using 200 snapshots from the last 100 ns trajectory of each system. Estimation of the binding free energy, represented by the sum of different energetic terms including Van der Waals (kJ/mol), electrostatic (kJ/mol), polar salvation (kJ/mol), and SASA (kJ/mol) energies, are shown in Table 2. The calculated binding free energy for complexes RBD-tomentin A, RBD-tomentin C, RBD-hyperoside, RBD-catechin gallate, and RBD-corylifol A were −28.72 ± 6.32, −22.94 ± 4.07, −16.20 ± 4.94, −22.12 ± 3.24, and −25.15 ± 6.22 kJ/mol, respectively. Per the general phenomena of the binding free energy calculations, a negative value always indicated a strong interaction and increased affinity between the protein and inhibitors at the atomic level. The observation showed that the complex of RBD-tomentin A (-28.72 ± 6.32 kJ/mol) has a higher negative binding affinity than the other considered complexes, while corylifol A (-25.15 ± 6.22 kJ/mol) demonstrated the second least binding energy. With a binding free energy value of −16.20 ± 4.94 kJ/mol, the RBD-hyperoside complex was determined to be the least stable one. The outcome of the binding free energy estimation is consistent with molecular docking and MDS (RMSD, Rg, and SASA) findings.

**Table 2.** Binding free energy of the complexes calculated using MM/PBSA approach using 200 snapshots from the last 100 ns trajectory of each system.

MD systems	van der Waal energy (kJ/mol)	Electrostatic energy (kJ/mol)	Polar solvation energy (kJ/mol)	SASA energy (kJ/mol)	Binding energy (kJ/mol)
Tomentin A	−84.07 ± 7.93	−74.21 ± 6.83	139.12 ± 11.73	−9.74 ± 0.82	−28.72 ± 6.32
Tomentin C	−86.76 ± 6.82	−92.57 ± 7.83	166.92 ± 14.42	−9.64 ± 0.81	−22.94 ± 4.07
Hyperoside	−76.64 ± 7.22	−77.95 ± 7.33	146.82 ± 13.83	−8.77 ± 0.81	−16.20 ± 4.94
Catechin gallate	−87.71 ± 6.78	−105.31 ± 8.31	183.24 ± 13.62	−10.03 ± 0.87	−22.12 ± 3.24
Corylifol A	−88.54 ± 8.06	−68.91 ± 7.06	137.38 ± 11.23	−9.05 ± 0.82	−25.15 ± 6.22

## 4. Conclusion

In this present study, a manually curated library of natural flavonoids derived from natural food sources (fruits, vegetables, etc.) was assessed for their inhibition potential against RBD of omicron (B.1.19) using a plethora of molecular modelling methods. Based on the molecular docking investigations, five flavonoids, specifically tomentin A, tomentin C, hyperoside, catechin gallate, and corylifol A, were ranked as the top interacting molecules with RBD. These screened molecules showed promising inhibition of RBD with binding affinity scores as (≥ 8.0 kcal/mol^−1^), and hydrogen bonds with mutated residues (≥ 2). MD simulations were performed to evaluate the structural stability of the top-ranked compounds at the atomic level in complex with RBD on 200 ns. The MD simulations and binding free energy analysis demonstrated the conformational stability pattern of these molecules with RBD throughout the simulation trajectories. Furthermore, PCA analysis revealed dominant motions and minor conformational changes in the complexes. Tomentin A occupied the largest conformational space, while catechin gallate, hyperoside, and corylifol A complexes displayed less motion. Additionally, the porcupine plots demonstrated mobility in the β-strand loop and outward motion at the terminal ends, indicating its significance in molecular recognition. Tomentin A, tomentin C, and corylifol A occupied larger conformational space with higher trace values, indicating their potential for versatile molecular interactions compared to catechin gallate and hyperoside complexes with RBD. Screened natural flavonoids in the present study harbour essential drug-likeness properties per Lipinski’s rule of five. Consequently, the present study suggests that five natural flavonoids, namely, tomentin A, tomentin C, hyperoside, catechin gallate, and corylifol A, have enough potential to inhibit the RBD of omicron in a significant manner. Further *in-vitro* and *in-vivo* experiments can be conducted for validation of these molecules. As many of the emerging omicron variants share similar mutation patterns within the RBD, with only one or two mutations differing, therefore, there is a possibility that these screened compounds may also demonstrate inhibition activity against other variants of the omicron lineage.

## Supplementary Material

Supplemental Material

## Data Availability

The original contributions presented in the study are included in the article/Supplementary Material; further inquiries can be directed to the corresponding author.
