# Supplementary material for: Inhibition potential of natural flavonoids against selected omicron (B.1.19) mutations in the spike receptor binding domain of SARS-CoV-2: a molecular modeling approach
Source: J Biomol Struct Dyn. 2023 Dec 19;43(2):1068–82. doi: 10.1080/07391102.2023.2291165 (PMC11716671; doi:10.1080/07391102.2023.2291165)
Supplement: Supplemental Material [file TBSD_A_2291165_SM8224.docx]

**Suppl Figures**

**Suppl. Figure 1**


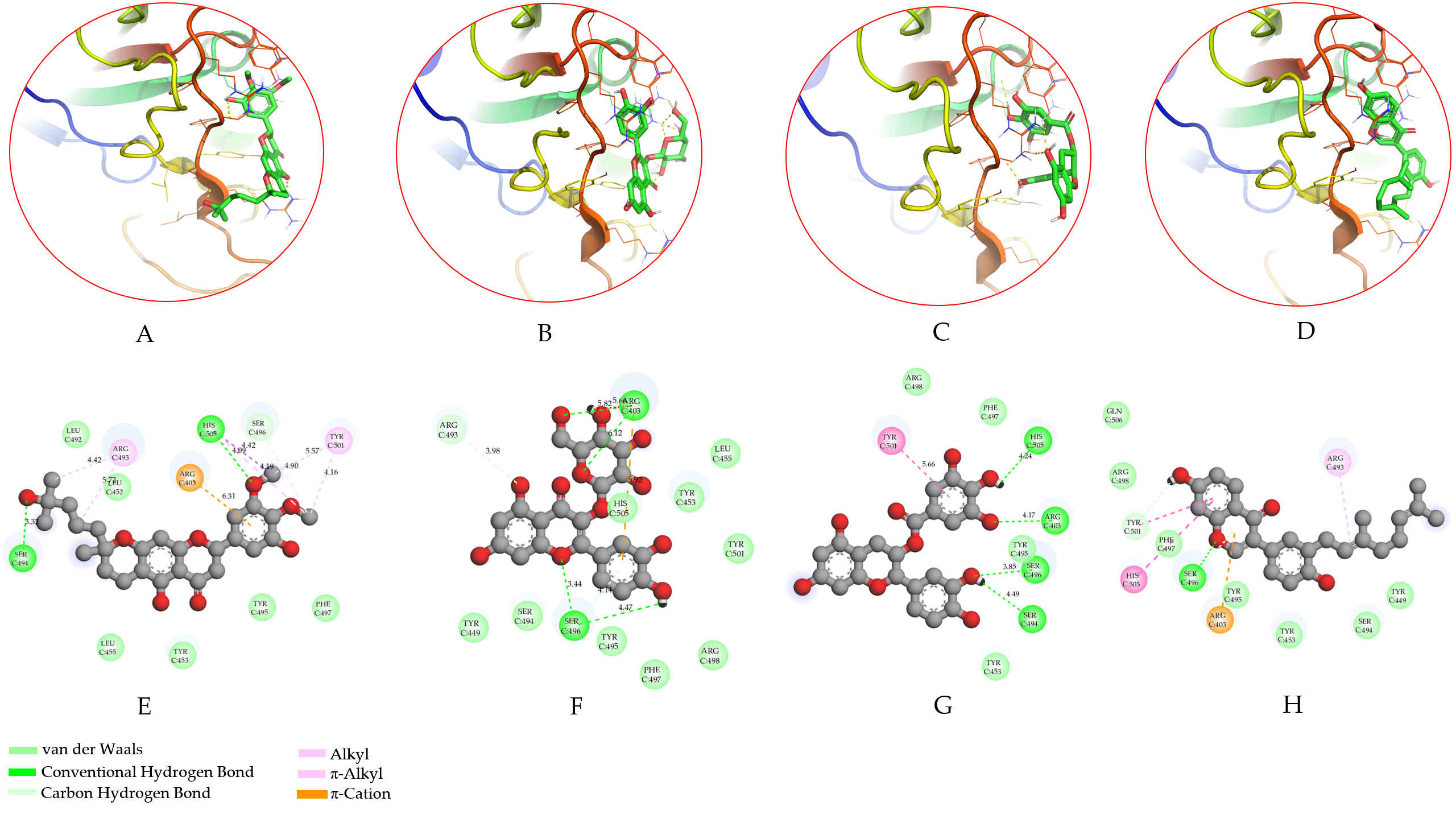


**Suppl Figure 1.** Representation of molecular interactions between RBD and tomentin C, hyperoside, catechin gallate, and corylifol A, in 3D and 2D views. **(A)** 3D binding mode of tomentin C with RBD. **(B)** 3D binding mode of hyperoside with RBD. **(C)** 3D binding mode of catechin gallate with RBD. **(D)** 3D binding mode of corylifol A with RBD. Compounds are shown in green sticks; RBD residues are depicted by atom-type color sticks; and hydrogen bonds are depicted with yellow dotted lines. 3D model of interactions was rendered using PyMOL; **(E)** 2D model of interaction between tomentin C and RBD. **(F)** 2D model of interaction between hyperoside and RBD. **(G)** 2D model of interaction between catechin gallate and RBD. **(H)** 2D model of interaction between corylifol A and RBD. Different types of interactions including conventional hydrogen bond, carbon hydrogen bond, van der Waals, alkyl, π alkyl, and π cation were annotated using the Discovery Studio program.

**Suppl. Figure 2**


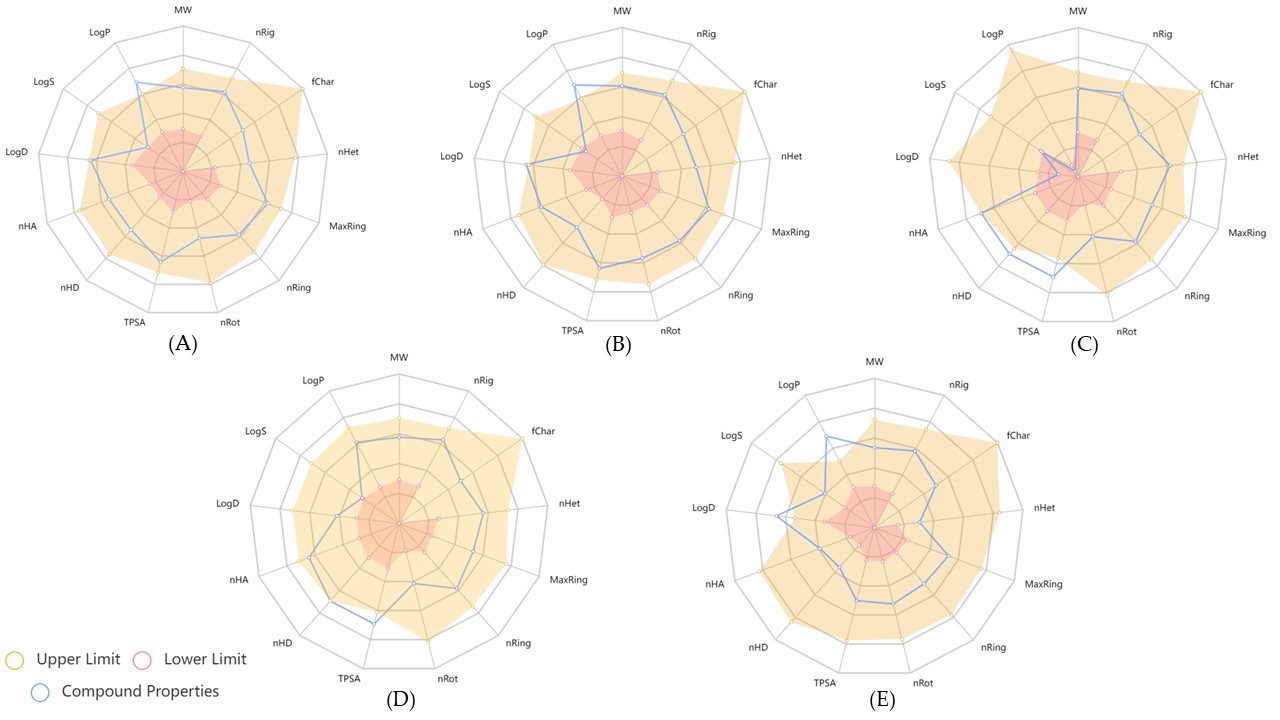


**Suppl. Figure 2.** Calculated graphs of drug-likeness properties; (A) Tomentin A, (B) Tomentin C, (C) Hyperoside, (D) Catechin gallate, and (E) Corylifol A.

**Supplementary Tables**

**Suppl. Table 1.** Virtual screening results of natural flavonoids against RBD of omicron along with ids, molecular weight, chemical formulas, 3D structure, binding energy, and molecular interactions.

| **S.N** | **CID** | **Name** | **Molecular Weight**  **(g/mol)** | **Chemical**  **formula** | **3D Structure** | **Binding**  **energy**  **(kcal/mol)** | **Molecular interactions** |
| --- | --- | --- | --- | --- | --- | --- | --- |
| 1 | 71659627 | Tomentin A | 442.5 | C_25_H_30_O_7_ | 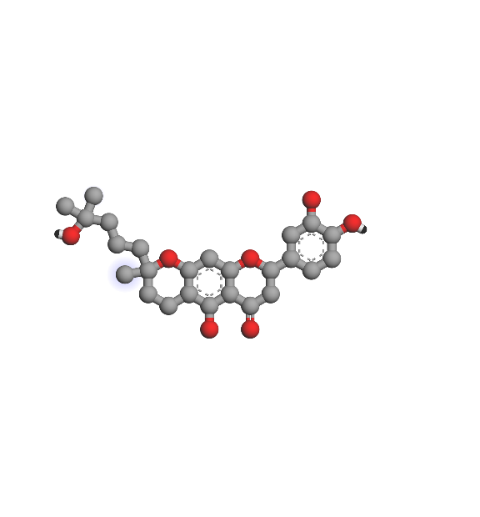 | -8.7 | **Hydrogen Bond**: Ser496 (3.56 Å), His505 (3.19 Å)  **Alkyl:** Arg493 (4.13, 5.55 Å)  **π**-**π Stacked:** Ser496 (3.97 Å), His505 (6.10 Å)  **van der Waals:** Arg403, Tyr495, Phe497, Arg498, Tyr501, Gln506 |
| 2 | 71659765 | Tomentin C | 486.6 | C_27_H_34_O_8_ | 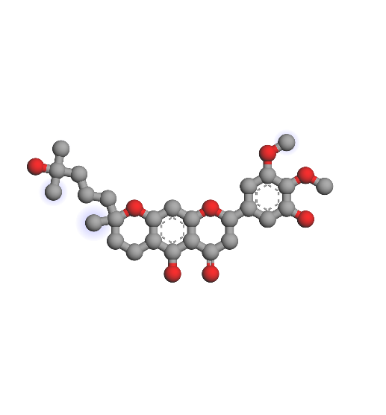 | -8.6 | **Hydrogen Bond:** Ser494 (3.32 Å), His505 (4.09 Å)  **Carbon Hydrogen:** Ser496 (4.90 Å)  **Alkyl:** Arg493 (4.42, 5.72 Å)  **π Alkyl:** Tyr501 (4.16 Å), His505 (4.09 Å)  **π** **Sigma**: His505 (4.42 Å)  **π** **Cation:** Arg403 (6.31 Å)  **van der Waals:** Tyr453, Leu455, Leu492, Tyr495, Ser496, Phe497 |
| 3 | 5281643 | Hyperoside | 464.4 | C_21_H_20_O_12_ | 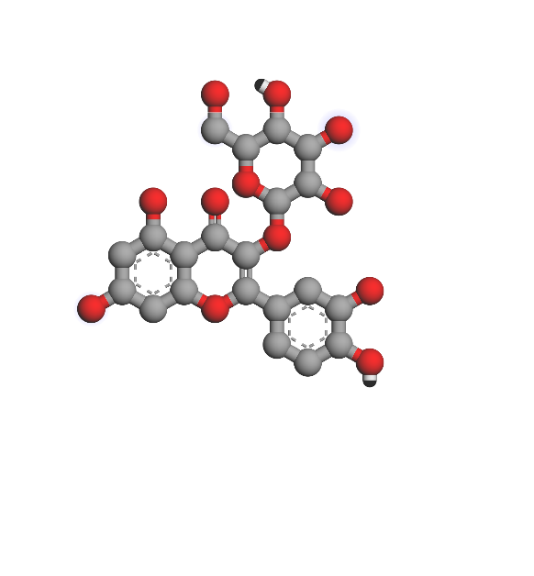 | -8.4 | **Hydrogen Bond**: Arg403 (5.82, 6.12 Å), Ser496 (3.44, 4.47 Å)  **Carbon Hydrogen Bond**: Arg493 (3.98 Å)  **π-Donor Hydrogen Bond**: Ser496 (4.14 Å)  **π-Cation**: Arg403 (5.92 Å)  **van der Waals**: Tyr449, Tyr453, Ser494, Leu455, Tyr495, Phe497, Arg498, Tyr501 |
| 4 | 6419835 | Catechin gallate | 442.4 | C_22_H_18_O_10_ | 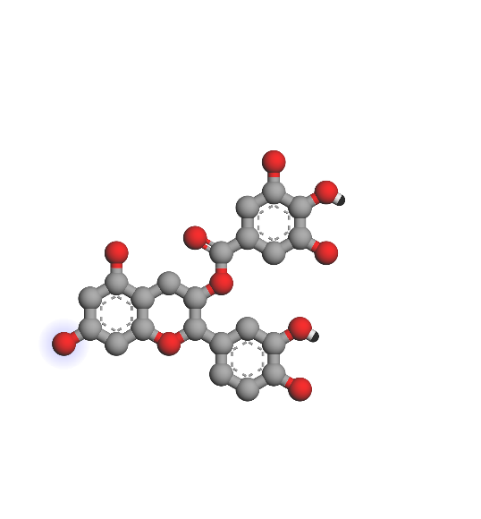 | -8.3 | **Hydrogen Bond**: Arg403 (4.17 Å), Ser494 (4.49 Å), Ser496 (3.85 Å), His505 (4.24 Å)  **π**-**π T-Shaped:** Tyr501 (5.66 Å)  **van der Waals:** Tyr453, Tyr495, Phe497, Arg498 |
| 5 | 25056407 | Corylifol A | 390.5 | C_25_H_26_O_4_ | 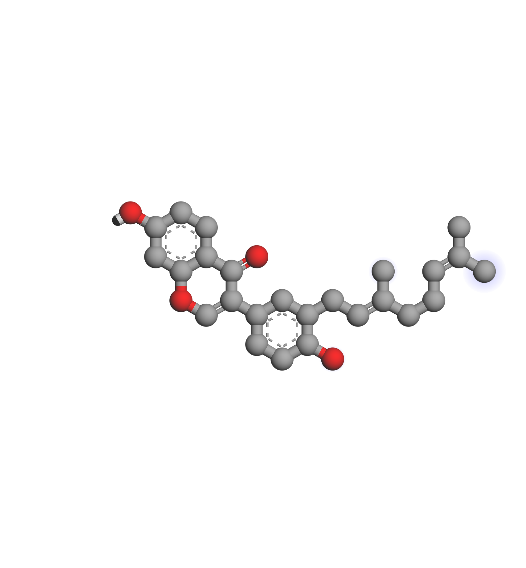 | -8.2 | **Hydrogen Bond**: Ser496 (4.09 Å)  **π Donor Hydrogen Bond:** Tyr501 (3.33 Å)  **Alkyl:** Arg493 (4.09 Å)  **π** **Cation:** Arg403 (6.13 Å)  **π**-**π T-Shaped:** Tyr501 (6.50 Å)  **π**-**π Stacked:** His505 (5.53 Å)  **van der Waals:** Tyr449, Tyr453, Ser494, Tyr495, Phe497, Arg498, Gln506 |
| 6 | 932 | Naringenin | 272.25 | C_15_H_12_O_5_ | 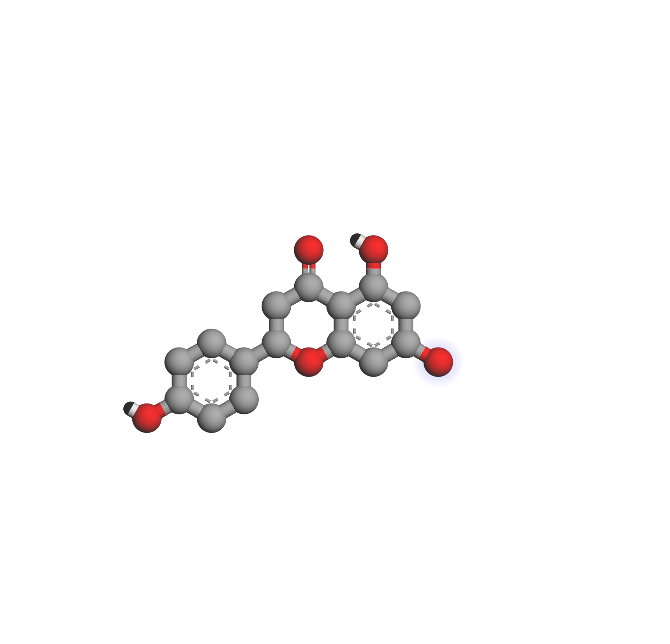 | -6.6 | **Hydrogen Bond**: Tyr453 (4.39), Ser496 (3.52), His505 (5.29)  **π-π T-Shaped**: Tyr501 (6.92)  **van der Waals**: Gln403, Leu455, **Arg493**, Ser494, Tyr495, Phe497, **Arg498**, Gln506, Pro507 |
| 7 | 9064 | Catechin | 290.27 | C_15_H_14_O_6_ | 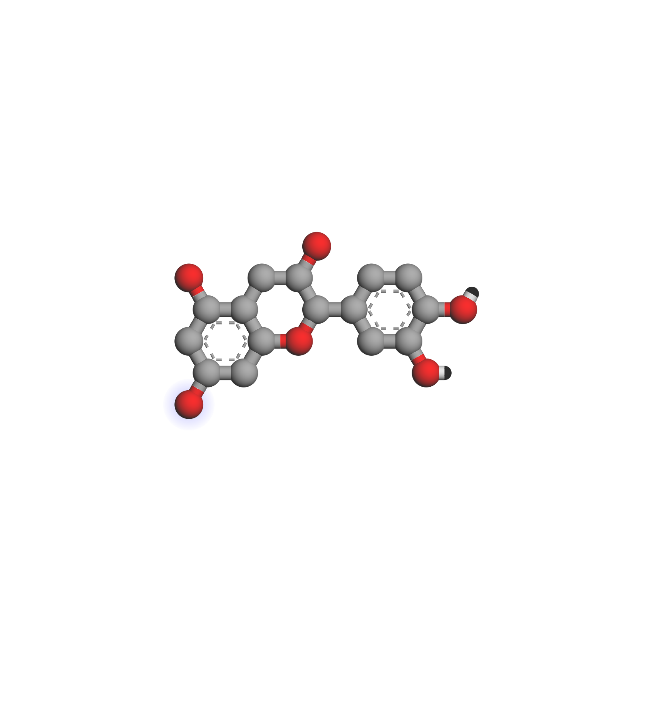 | -6.6 | **Hydrogen Bond**: **Ser496** (3.39), His505 (3.55)  **π-Donor Hydrogen Bond**: His505 (3.47)  **π-Alkyl**: **Arg493** (5.89)  **π-π Stacked**: **Ser496** (3.93)  **Amide π Stacked**: **His505** (6.11)  **van der Waals**: Arg403, Tyr449, Leu455, Ser494, Tyr495, Phe497, Arg498, Tyr501, Gln506 |
| 8 | 10621 | Hesperidin | 610.6 | C_28_H_34_O_15_ | 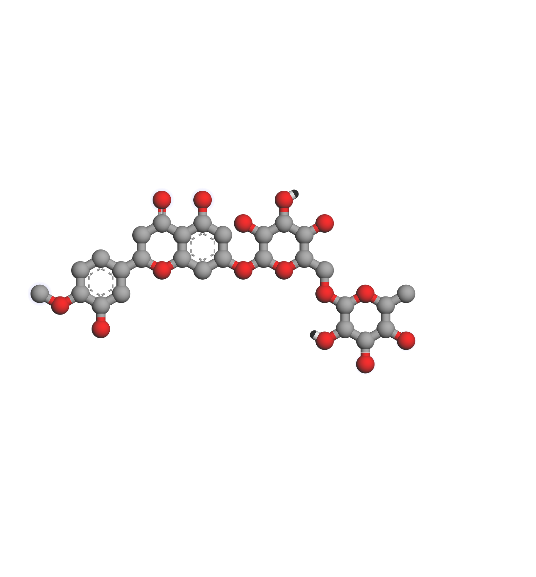 | -8.1 | **Hydrogen Bond**: Phe342 (6.36), Asn343 (3.63), Trp436 (5.25), Lys440 (3.62)  **π-Cation**: Lys440 (5.50)  **Alkyl**: Leu368 (3.80), Leu371 (5.44), **Lys440** (4.04)  **π-Alkyl**: Pro373 (4.84), Phe374 (6.80)  **van der Waals**: Phe338, Asp339, Val367, Ser438, Asn439, Phe375, Asn437, Lu441, Arg509 |
| 9 | 65064 | Epigallocatechin Gallate | 458.4 | C_22_H_18_O_11_ | 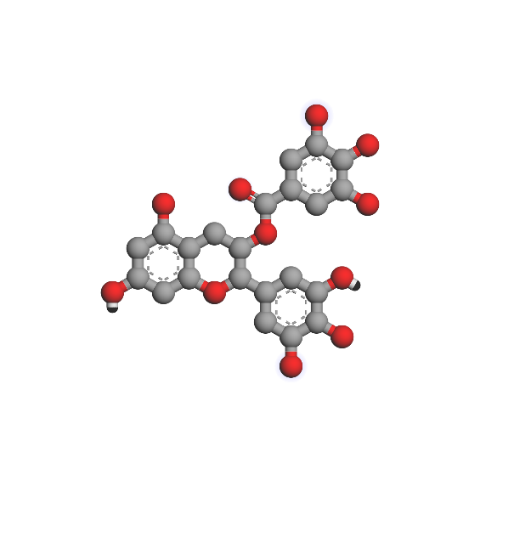 | -7.2 | **Hydrogen Bond**: Phe342 (6.83), Ala344 (4.25), Arg509 (5.02)  **π-Alkyl**: Trp436 (6.35)  **π-Sigma**: Leu441 (5.61)  **van der Waals**: Pro373, Phe374, Phe375, Asn437, Asn439, Ser438 |
| 10 | 72277 | Epigallocatechin | 306.27 | C_15_H_14_O_7_ | 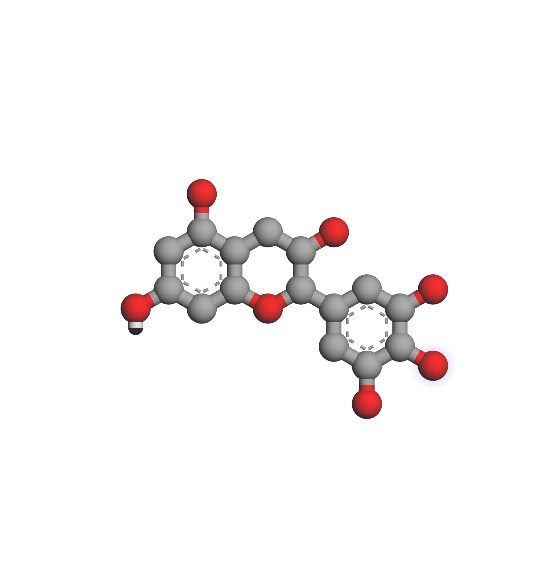 | -6.1 | **Hydrogen Bond**: Asn343 (3.74)  **π-π T-Shaped**: Trp436 (6.83)  **Alkyl**: Leu441 (5.47)  **π-Alkyl**: **Pro373** (5.01), Trp436 (5.43)  **van der Waals**: Phe342, Ala344, Phe375, Asn437, Ser438, Lys440, Arg509 |
| 11 | 72281 | Hesperetin | 302.28 | C_16_H_14_O_6_ | 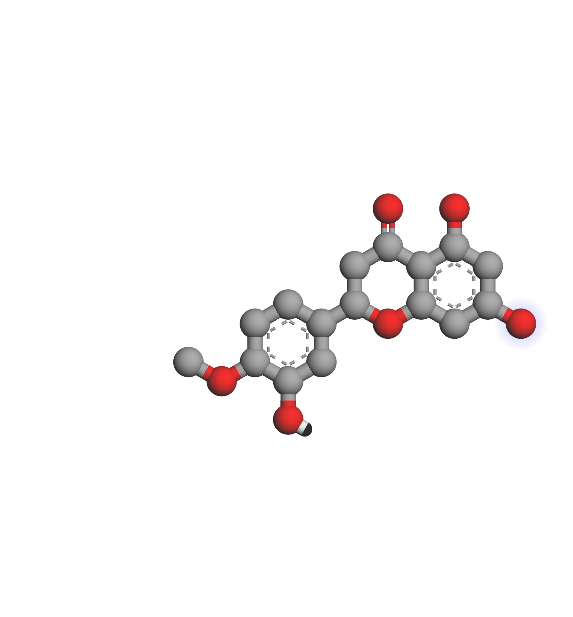 | -6.5 | **Hydrogen Bond**: Phe342 (5.89)  **π-Sigma**: Val367 (5.46)  **π-Anion**: Asp339 (5.21)  **Alkyl**: **Pro373** (4.51)  **π-Alkyl**: Phe374 (5.29), Trp436 (5.10)  **van der Waals**: Phe338, Asn343, Leu368, Leu371 |
| 12 | 128861 | Cyanidin | 287.24 | C_15_H_11_O_6_^+^ | 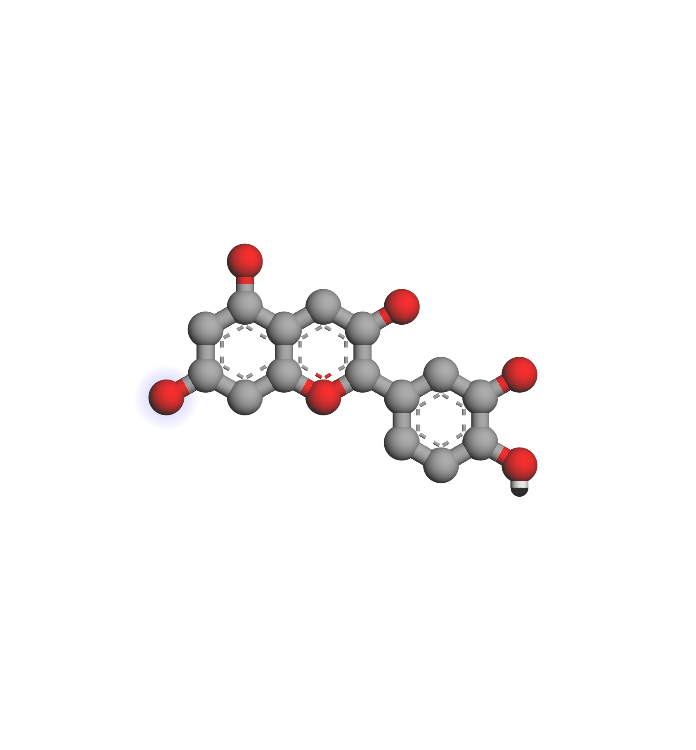 | -6.5 | **π-Donor Hydrogen Bond**: Ser496 (3.44), **Tyr501** (3.07)  **π-Alkyl**: Arg493 (5.86)  **π-π Stacked**: Ser496 (3.80)  **Amide Pi-Stacked**: His505 (6.19)  **van der Waals**: Arg403, Tyr449, Tyr453, Leu455, Ser494, Tyr495, Phe497, Arg498 |
| 13 | 168849 | Pectolinarin | 622.6 | C_29_H_34_O_15_ | 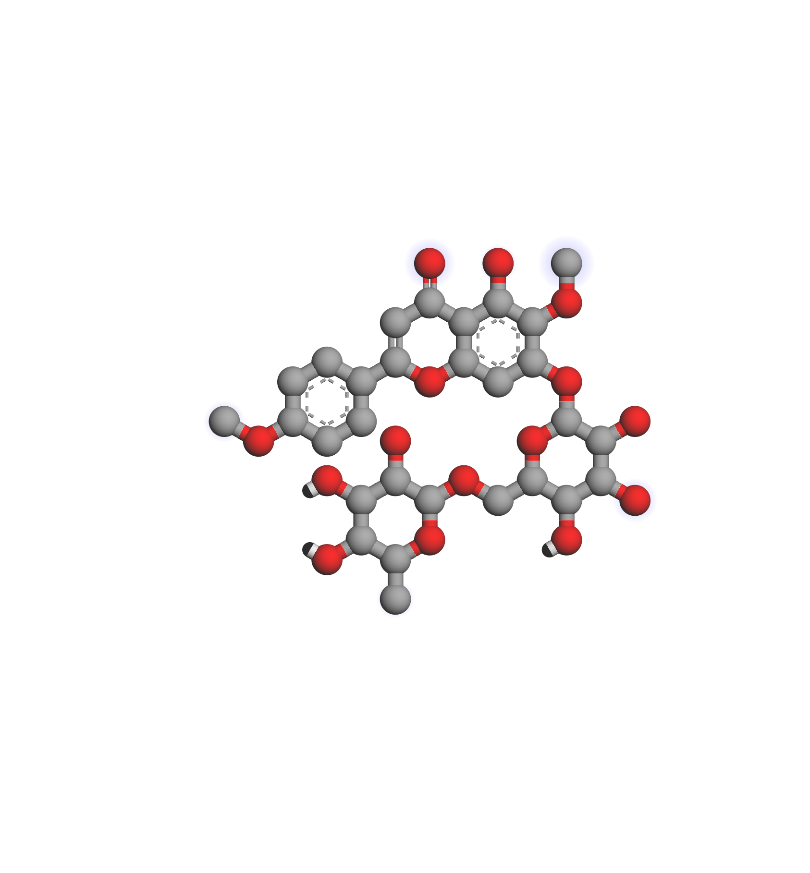 | -7.0 | **Hydrogen Bond**: Gln409 (5.44), Tyr453 (7.02), Ser496 (4.18, 4.36), His505 (3.67, 3.70)  **π-Donor Hydrogen Bond**: Tyr501 (4.10)  **π-Anion**: Asp405 (6.55)  **π-Cation:** Arg403 (5.85, 7.93)  **van der Waals**: Glu406, Arg408, Asn417, Tyr449, Arg493, Ser494, Tyr495, Phe497 |
| 14 | 442428 | Naringin | 580.5 | C_27_H_32_O_14_ | 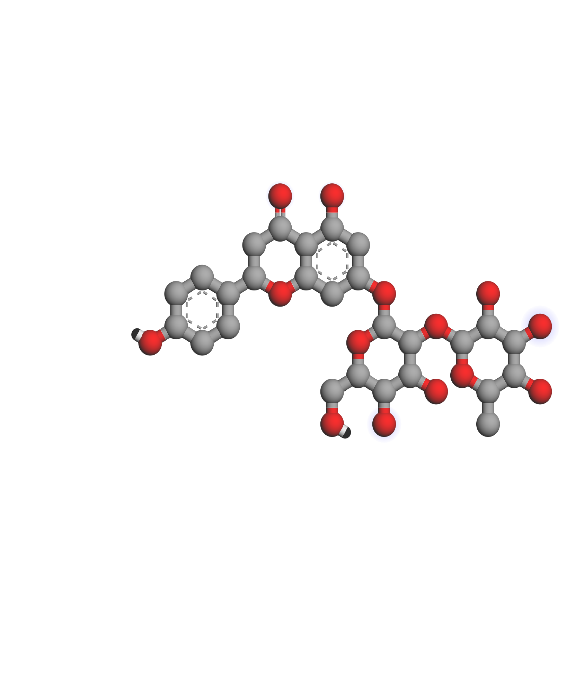 | -6.8 | **Hydrogen Bond**: Ser494 (3.15, 3.18), **Arg498** (6.60), His505 (4.04)  **Carbon Hydrogen Bond**: Phe497 (4.24)  **π-π Stacked**: Tyr501 (7.21)  **π-π T-Shaped**: His505 (5.63)  **van der Waals**: Arg403, Tyr449, Arg493, Tyr495, Ser496, Gln506 |
| 15 | 442439 | Neohesperidin | 610.6 | C_28_H_34_O_15_ | 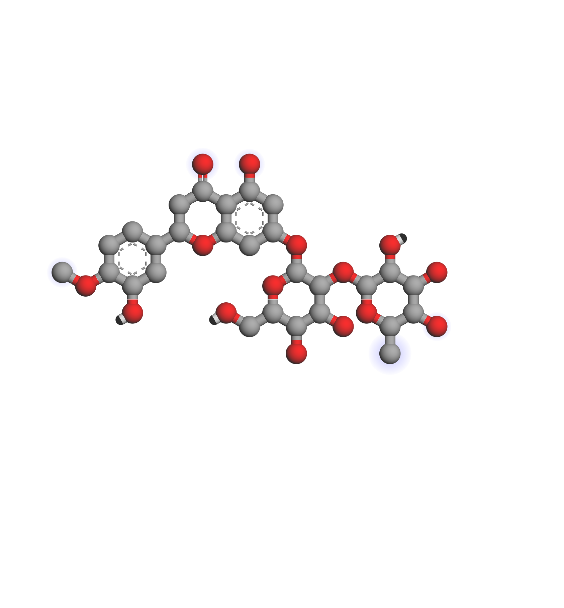 | -6.5 | **Hydrogen Bond**: Arg403 (5.42), Tyr453 (6.24), Ser496 (3.78, 3.82), His505 (3.56)  **Carbon Hydrogen Bond**: Tyr495 (4.11), **Ser496** (3.87), Thr500 (5.05)  **π-π Stacked**: Tyr501 (5.05)  **π-π T-Shaped**: His505 (5.06)  **van der Waals**: Arg493, Ser494, Phe497, Arg498, Gln506 |
| 16 | 5276890 | Gallocatechin Gallate | 458.4 | C_22_H_18_O_11_ | 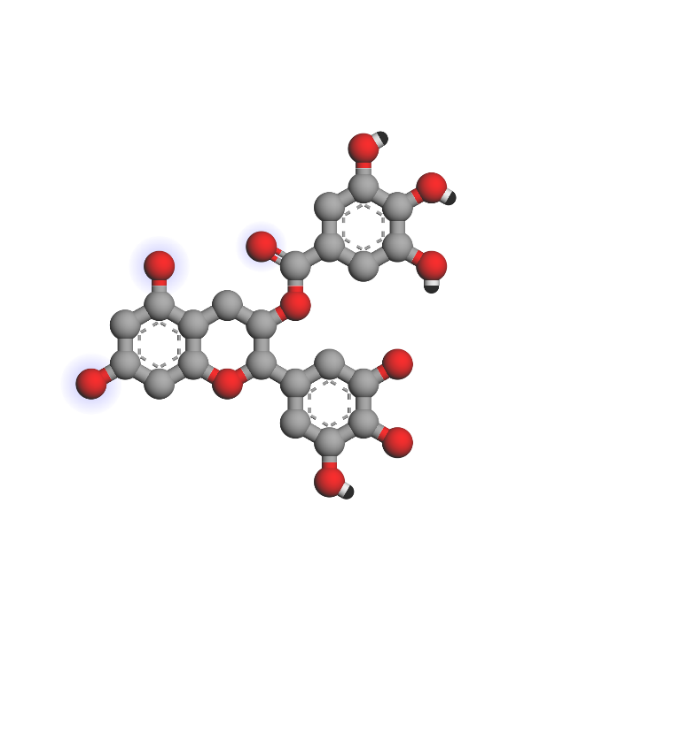 | -6.5 | **Hydrogen Bond**: Tyr453 (5.56), Ser494 (4.75), His505 (4.90)  **π-Alkyl**: Tyr501 (6.67), His505 (6.23)  **π-π Stacked**: Tyr501 (5.29, 6.58)  **π-π T-Shaped**: His505 (4.56)  **van der Waals**: Arg403, Arg493, Arg498, Tyr495, Gln506, Pro507 |
| 17 | 5280343 | Quercetin | 302.23 | C_15_H_10_O_7_ | 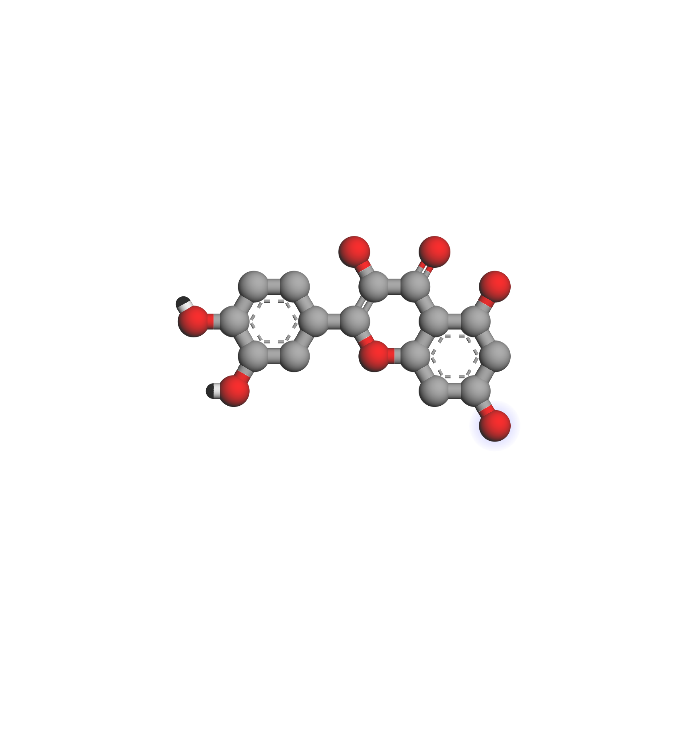 | -6.3 | **Hydrogen Bond**: Ser496 (3.39), His505 (4.04)  **π-Donor Hydrogen Bond**: Arg493 (4.14)  **Carbon Hydrogen Bond**: Tyr501 (3.23)  **π-π Stacked**: His505 (6.19)  **Amide Pi Stacked**: Ser496 (3.88)  **van der Waals**: Arg403, Tyr449, Ser494, Phe497, Arg498, Gln506 |
| 18 | 5280443 | Apigenin | 270.24 | C_15_H_10_O_5_ | 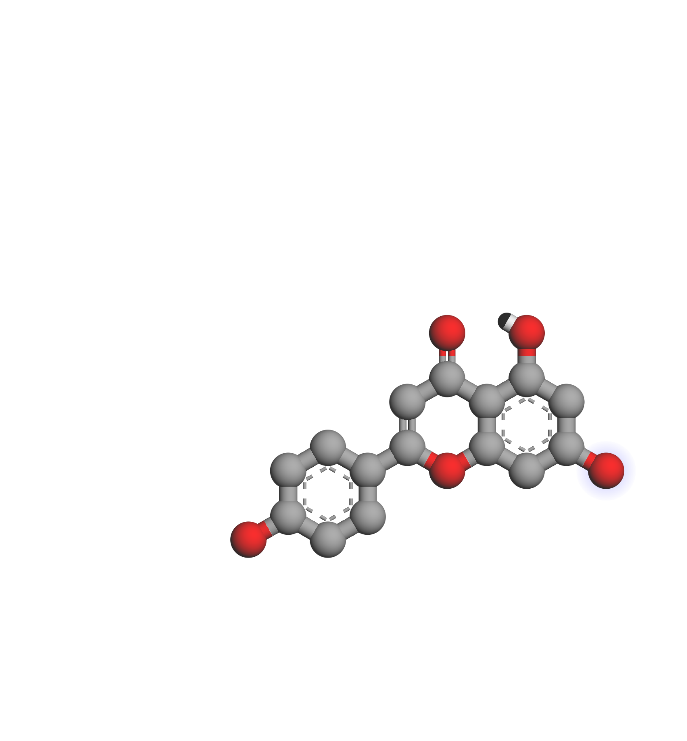 | -6.7 | **Hydrogen Bond**: Ser496 (3.51)  **π-π T-Shaped**: Tyr501 (7.18)  **π Alkyl**: **Arg493** (6.03)  **van der Waals**: Arg403, Leu455, Ser494, Tyr495, Phe497, Arg498 |
| 19 | 5280445 | Luteolin | 286.24 | C_15_H_10_O_6_ | 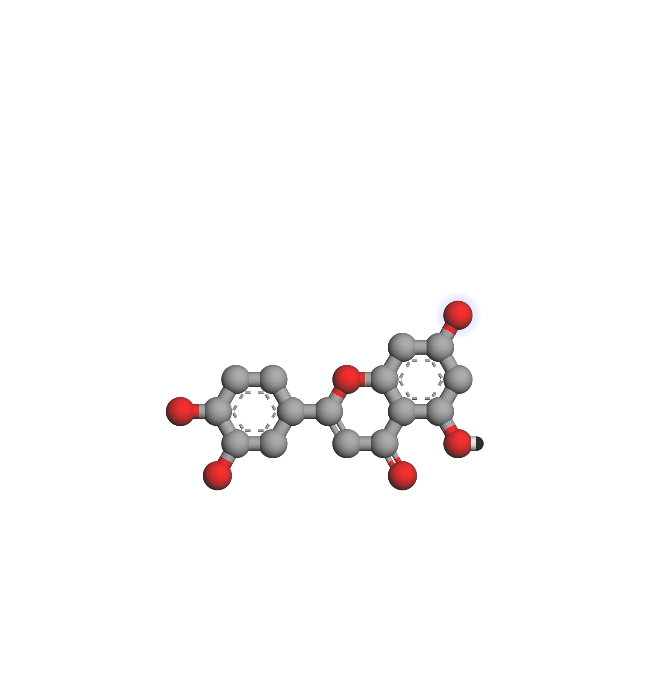 | -6.4 | **Hydrogen Bond**: Tyr453 (5.28), Ser496 (3.59)  **π-π T-Shaped**: His505 (6.15)  **Amide π Stacked**: Ser496 (3.92)  **π Alkyl**: Arg493 (5.98)  **van der Waals**: Arg403, Tyr449, Leu455, Ser494, Tyr495, Phe497, Arg498, Tyr501 |
| 20 | 5280544 | Herbacetin | 302.23 | C_15_H_10_O_7_ | 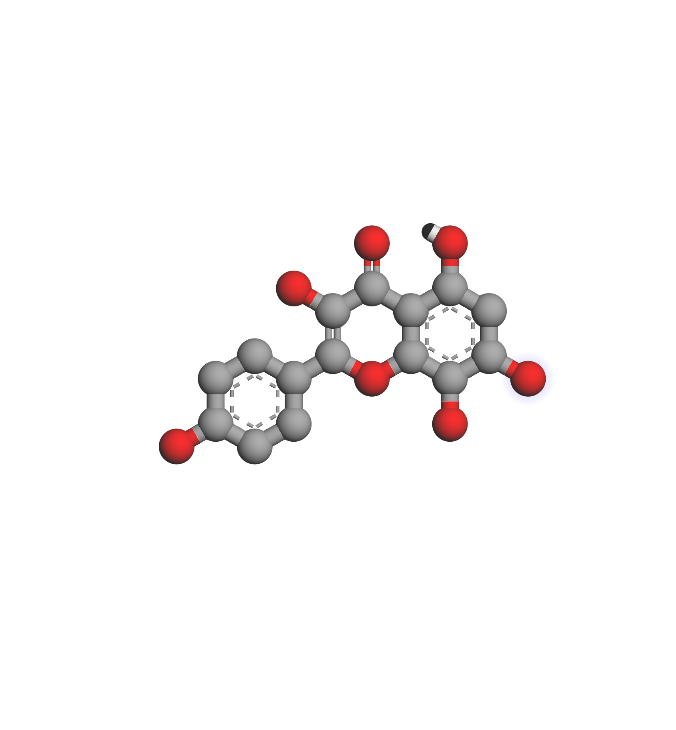 | -6.5 | **Hydrogen Bond**: Tyr453 (5.66), Ser496 (3.60)  **π-π T-Shaped**: Tyr501 (7.38)  **π Alkyl**: Arg493 (5.79)  **van der Waals**: Arg403, Tyr449, Leu455, Ser494, Tyr495, Phe497, Arg498, His505 |
| 21 | 5280863 | Kaempferol | 286.24 | C_15_H_10_O_6_ | 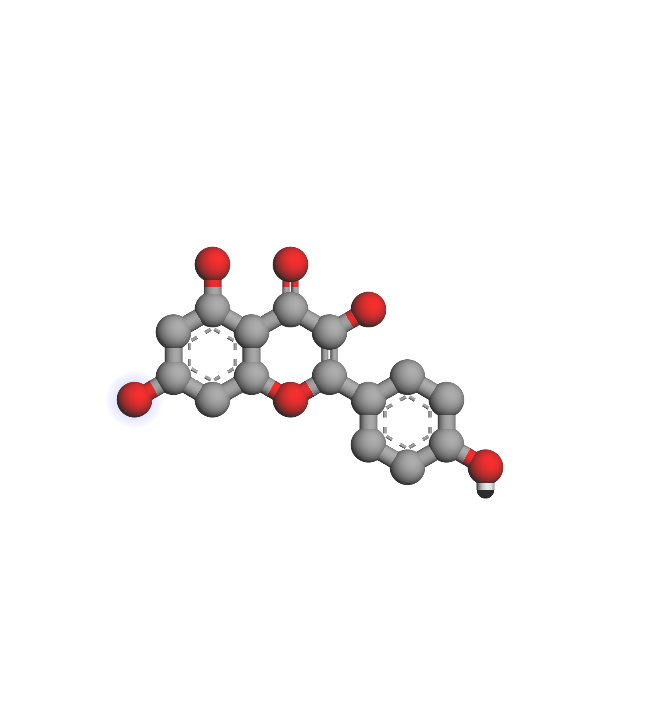 | -6.5 | **Hydrogen Bond**: Arg403 (5.06), Ser496 (3.39, 4.99)  **π Alkyl**: Arg493 (5.96)  **van der Waals**: Tyr449, Leu455, Ser494, Tyr495, Phe497, Arg498, Tyr501 |
| 22 | 5280961 | Genistein | 270.24 | C_15_H_10_O_5_ | 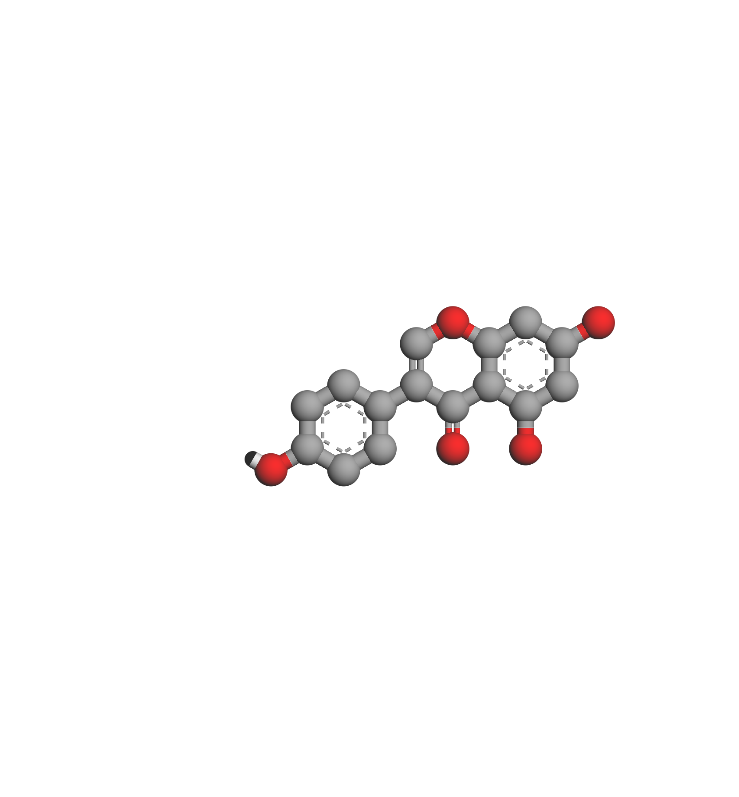 | -6.6 | **Hydrogen Bond**: Ser496 (3.40), His505 (4.38)  **π-π T-Shaped**: Tyr501 (7.19)  **van der Waals**: Tyr 453, Leu455, Arg493, Ser494, Tyr495, Phe497, Gln506, Pro507 |
| 23 | 5281255 | Isobavachalcone | 324.4 | C_20_H_20_O_4_ | 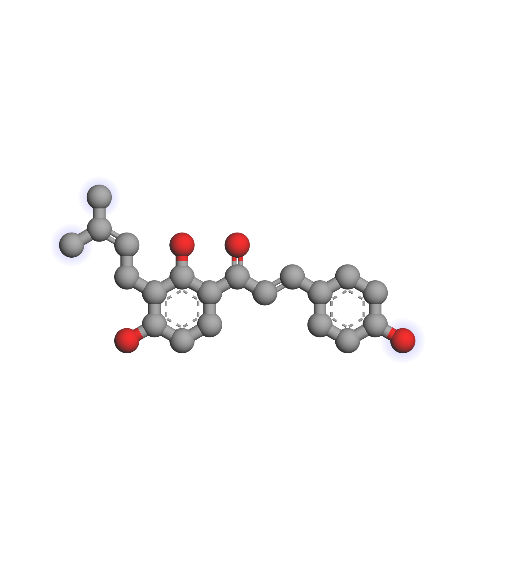 | -6.0 | **Hydrogen Bond**: Arg403 (5.51, 5.73)  **π Alkyl**: Arg403 (5.51), Arg493 (5.960, Tyr501 (6.22)  **van der Waals**: Tyr449, Tyr453, Ser494, Tyr495, Ser496, Phe497, His505 |
| 24 | 5281600 | Amentoflavone | 538.5 | C_30_H_18_O_10_ | 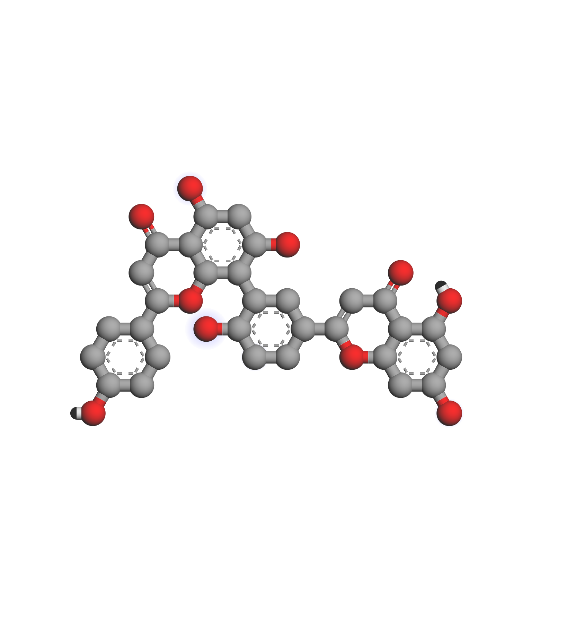 | -7.8 | **Hydrogen Bond**: Arg403 (6.46), Glu406 (4.15), Asn417 (4.20), Arg493 (5.67)  **π-Cation**: Arg403(5.86, 6.27, 6.96)  **van der Waals**: Asp405, Gln409, Ile418, Tyr449, Leu455, Tyr453, Ser494, Tyr495, Ser496 |
| 25 | 5281672 | Myricetin | 318.23 | C_15_H_10_O_8_ | 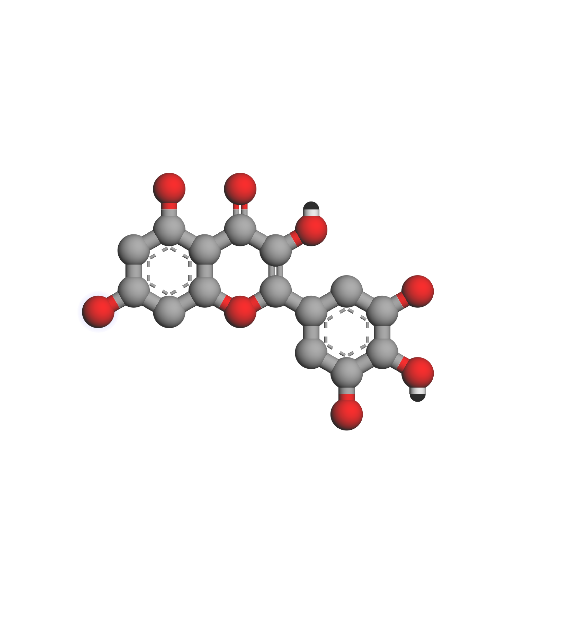 | -6.2 | **Hydrogen Bond**: Tyr453(5.90), Ser496 (3.55), His505 (3.20, 3.37, 4.57)  **Carbon Hydrogen Bond**: Arg493 (3.68, 3.96)  **π-Cation**: Arg403 (6.12)  **van der Waals**: Tyr449, Ser494, Tyr495, Phe497 |
| 26 | 5281697 | Scutellarein | 286.24 | C_15_H_10_O_6_ | 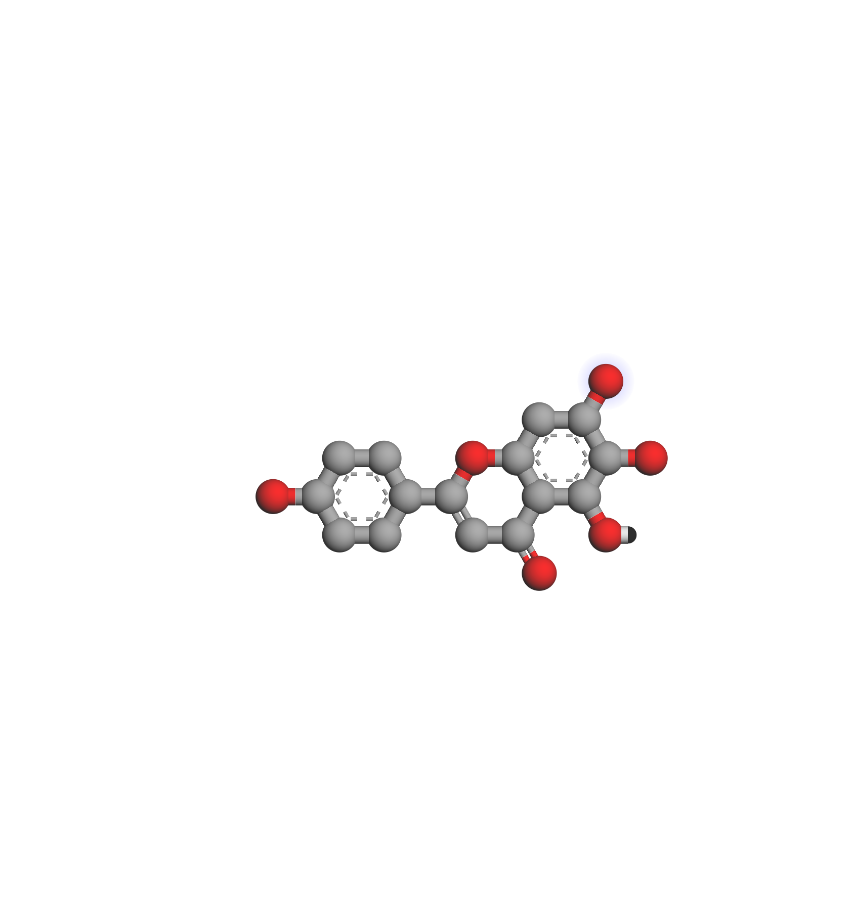 | -6.5 | **Hydrogen Bond**: Tyr453 (5.33), Arg493 (3.71), Ser496 (3.51)  **π-π Stacked**: Tyr501 (7.06)  **π-π T-Shaped**: His505 (5.99)  **van der Waals**: Arg403, Leu455, Ser494, Tyr495, Phe497, Arg498, Gln506, Pro507 |
| 27 | 5281806 | Psoralidin | 336.3 | C_20_H_16_O_5_ | 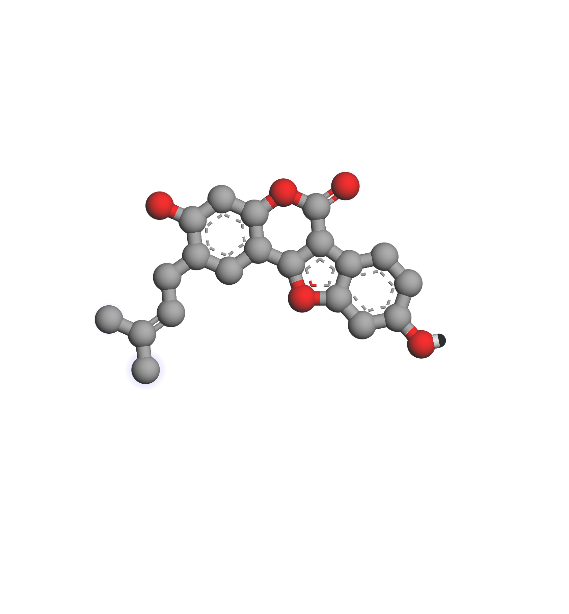 | -6.5 | **Hydrogen Bond**: Arg403 (6.30), Ser496 (3.75, 4.07)  **Alkyl**: Arg493 (4.62)  **π-Alkyl**: Tyr449 (5.51)  **van der Waals**: Ser494, Tyr495, Phe497, Arg498 |
| 28 | 5282150 | Rhoifolin | 578.5 | C_27_H_30_O_14_ | 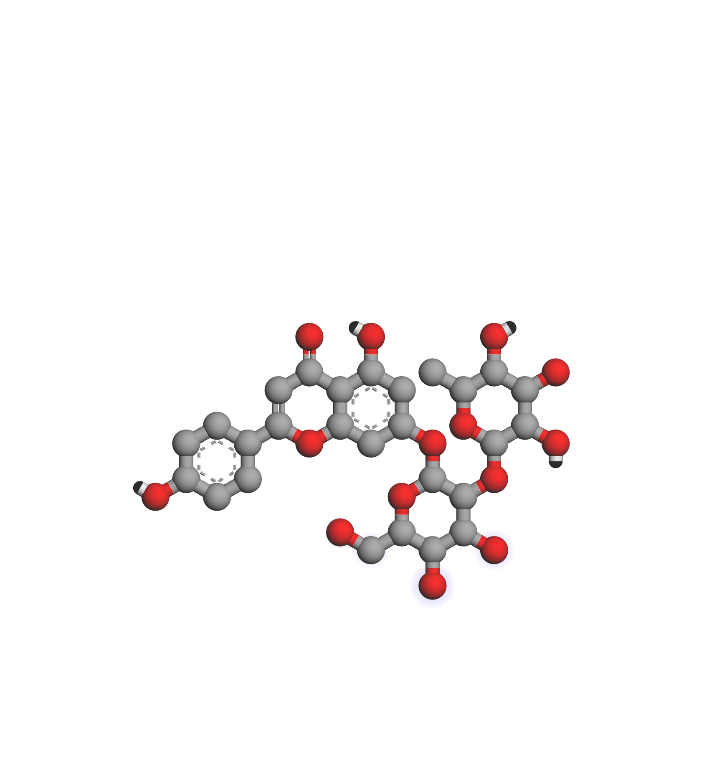 | -7.0 | **Hydrogen Bond**: Arg403 (5.43,5.87), Asp405 (5.02), Gl406 (4.44), Gln409 (5.90), Asn417 (3.60), Tyr453 (5.69)  **π-Alkyl**: Tyr453 (5.69)  **π-π Stacked**: Tyr501 (7.46)  **π-π T-Shaped**: His505 (6.06)  **π-Cation**: Arg403 (5.49)  **van der Waals**: Ile418, Leu455 Arg493, Ser494, Tyr495, Phe497, Arg498 |
| 29 | 5318997 | Icariin | 676.7 | C_33_H_40_O_15_ | 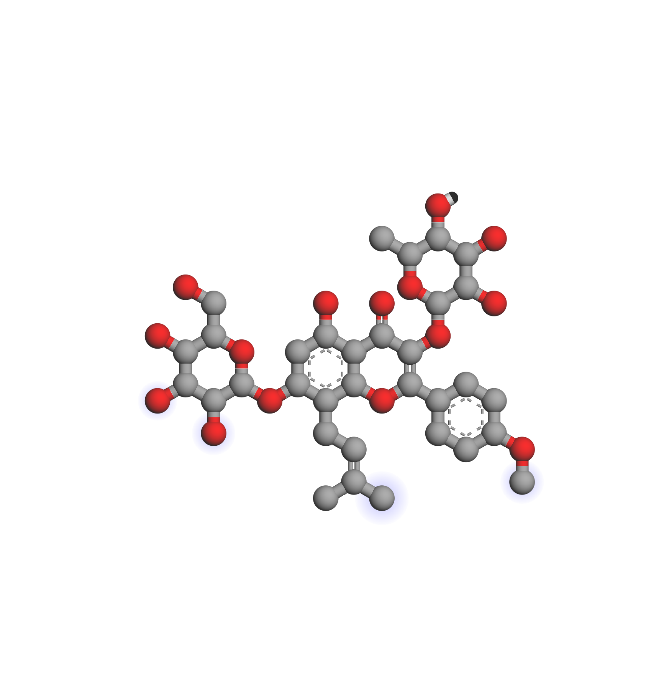 | -6.7 | **Hydrogen Bond**: Arg403 (4.51), Asn417 (4.95)  **Carbon Hydrogen Bond**: Tyr501 (3.94)  **π-Donor Hydrogen Bond**: Glu406 (4.85)  **π-Sigma**: His505 (4.33)  **π-Cation**: Arg403 (7.52)  **Alkyl**: Leu455 (4.86, 5.96)  **π-Alkyl**: Arg493 (6.20), Tyr501 (5.54)  **van der Waals**: Arg408, Gln409, Gly416, Ile418, Tyr453, Ser496, Tyr495, Ser494 |
| 30 | 5320053 | Neobavaisoflavone | 322.4 | C_20_H_18_O_4_ | 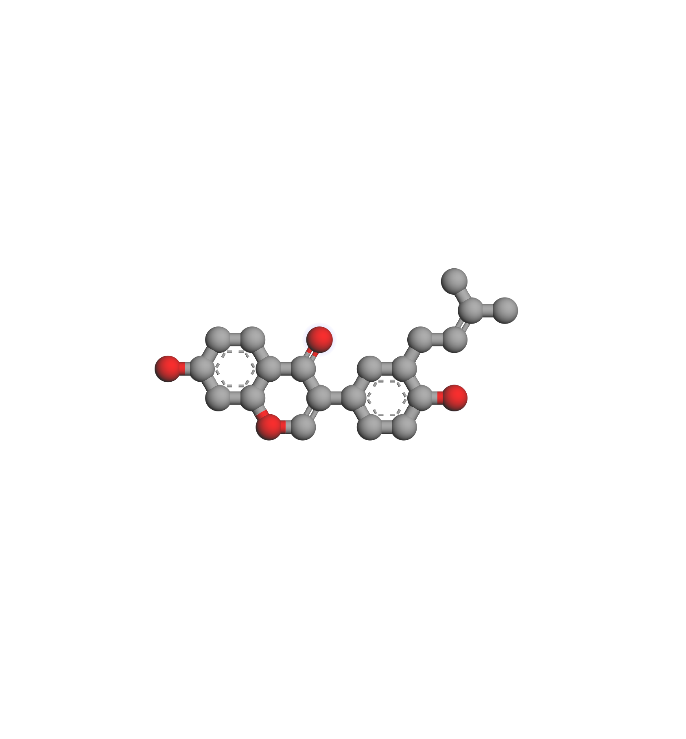 | -6.3 | **π**-**Alkyl**: Tyr453 (4.95)  **π**- **π**-**Stacked**: His505 (5.57)  **π**-**Cation**: Arg403 (5.36)  **van der Waals:** Glu406, Asn417, Ile418, Leu455, Tyr495, Ser496, Phe497, Arg498, Tyr501 |
| 31 | 5321765 | 4'-O-Methylbroussochalcone B | 338.4 | C_21_H_22_O_4_ | 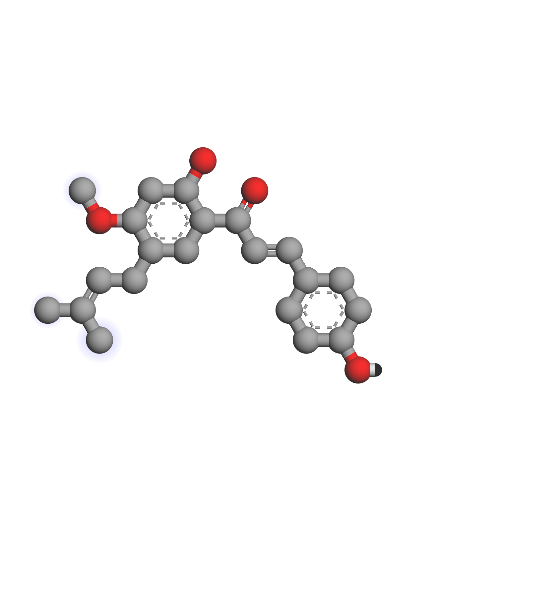 | -5.9 | **Hydrogen Bond**: His505 (4.60)  **π**- **π**-**Stacked**: Tyr501 (7.15)  **π**- **π T-Shaped:** His505 (5.66)  **van der Waals:** Arg403, Tyr449, Tyr453, Leu455, Arg493, Ser494, Tyr495, Ser496, Phe497, Arg498, Gln506, Pro507 |
| 32 | 6253344 | Helichrysetin | 286.28 | C_16_H_14_O_5_ | 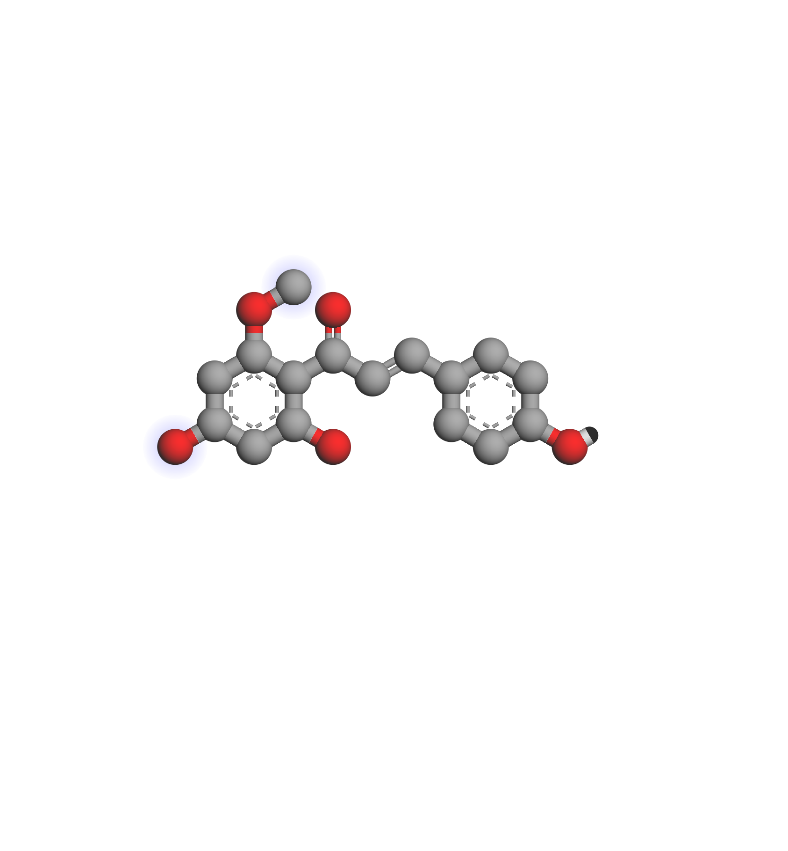 | -5.7 | **π**-**Donor Hydrogen Bond**: Tyr501 (3.21)  **π**-**Alkyl**: Arg493 (5.88)  **π**- **π**-**Stacked**: Tyr501 (7.05)  **π**- **π T-Shaped**: His505 (5.66)  **van der Waals:** Arg403, Tyr453, Ser494, Tyr495, Ser496, Phe497, Arg498 |
| 33 | 10022050 | Xanthoangelol E | 370.4 | C_21_H_22_O_6_ | 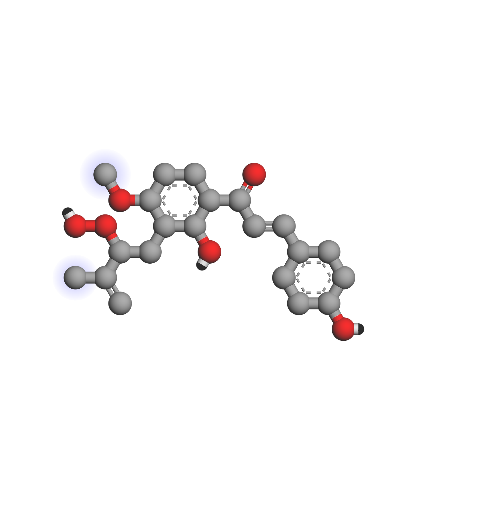 | -6.1 | **Hydrogen Bond**: Ser494 (2.73, 3.99), Ser496 (4.70)  **π-Alkyl:** Tyr449 (5.45)  **π**-**π T-Shaped:** Tyr501 (7.15)  **π**-**π Stacked:** His505 (4.48)  **van der Waals:** Arg403, Leu452, Tyr453, Arg493, Tyr495, Phe497 |
| 34 | 10337211 | Bavachinin | 338.4 | C_21_H_22_O_4_ | 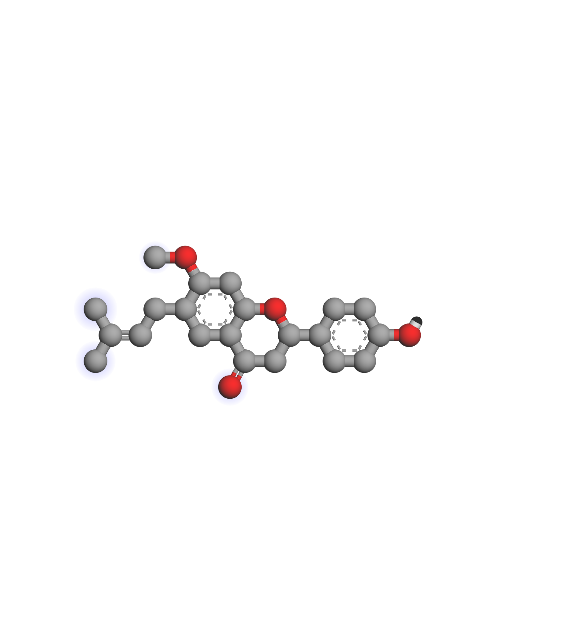 | -6.2 | **Hydrogen Bond**: **His505** (4.97)  **Carbon Hydrogen Bond**: Ser494 (5.24)  **Alkyl**: Leu455 (5.27), Arg493 (4.01)  **π**-**π T-Shaped:** Tyr501 (6.84)  **π**-**π Stacked:** His505 (5.41)  **van der Waals:** Arg403, Tyr453, Tyr495, Ser496, Phe497, Arg498, Gln506, Pro507 |
| 35 | 10343070 | Broussonol E | 438.5 | C_25_H_26_O_7_ | 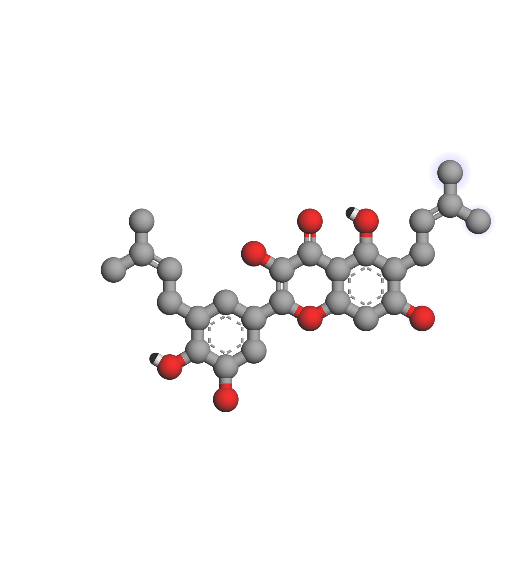 | -6.6 | **Hydrogen Bond**: Tyr449 (5.49), Ser494 (4.85)  **π Sigma:** Tyr449 (4.40)  **Alkyl:** Arg403 (5.19)  **π Alkyl:** Tyr495 (4.57), Phe497 (4.20), **Tyr501** (4.94), His505 (4.26)  **van der Waals:** Tyr453, Leu455, Ser496 |
| 36 | 44562555 | Caflanone | 368.4 | C_21_H_20_O_6_ | 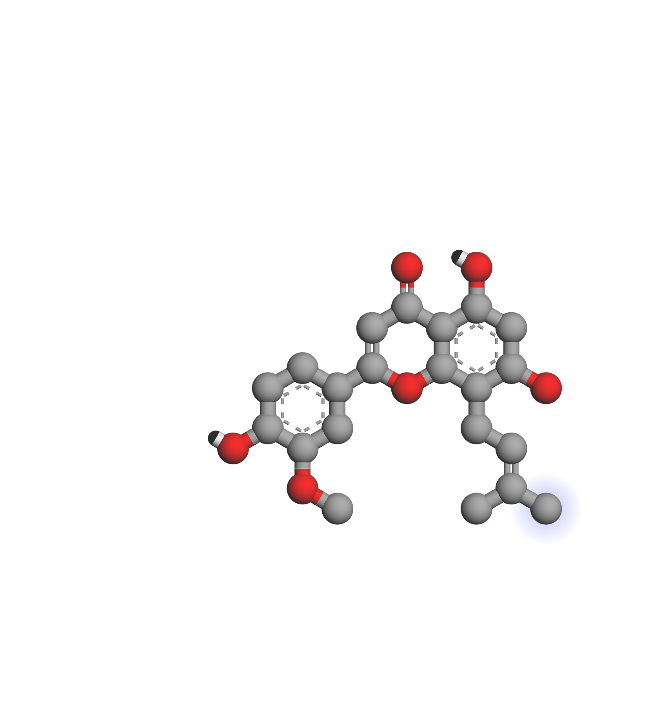 | -6.6 | **Hydrogen Bond**: Ser496 (4.06), His505 (3.36)  **π Alkyl:** Ty449 (6.01), Arg493 (6.08), Tyr501 (4.94)  **π** **Sigma**: His505 (4.18)  **π**-**π Stacked:** His505 (6.33)  **Amide** **π Stacked:** Ser496 (4.24)  **π** **Cation:** Arg403 (5.59)  **van der Waals:** Tyr453, Leu455, Ser494, Tyr495, Phe497, Arg498, Gln506 |
| 37 | 71659628 | Tomentin B | 456.5 | C_26_H_32_O_7_ | 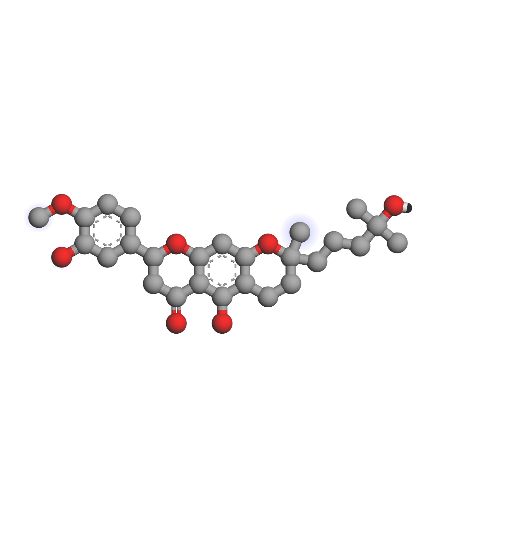 | -6.4 | **Hydrogen Bond**: Ser496 (4.14, 4.61)  **Alkyl:** Arg403 (3.93)  **π Alkyl:** Tyr449 (4.59), Arg493 (5.18), Tyr495 (4.72), Phe497 (4.96), Tyr501 (5.44), His505 (3.71)  **van der Waals:** Tyr453, Ser494, Arg498 |
| 38 | 71659766 | Tomentin D | 486.6 | C_27_H_34_O_8_ | 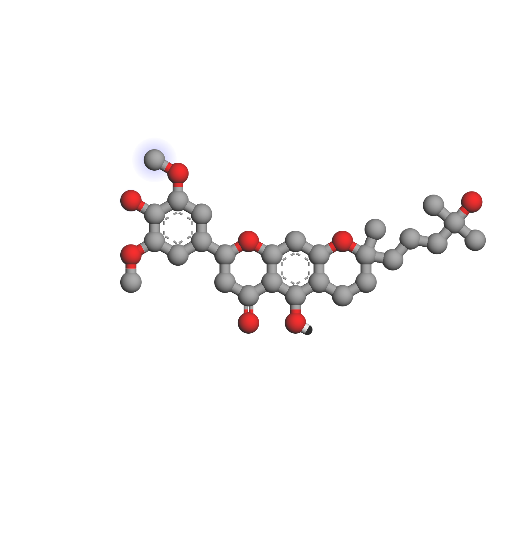 | -7.2 | **Hydrogen Bond:** Phe342 (6.13), Asn343 (3.43), Trp436 (5.35)  **Alkyl:** Val367 (4.35, 5.61), Leu368 (4.67, 4.80), Leu371 (5.21, 5.64), Lys440 (3.74)  **π Alkyl:** Pro373 (6.52), Phe374 (6.02), Lys440 (5.27)  **van der Waals:** Phe338, Asp339, Phe375, Asn437, Ser438, Asn439, Leu441, Arg509 |
| 39 | 71659767 | Tomentin E | 472.5 | C_26_H_32_O_8_ | 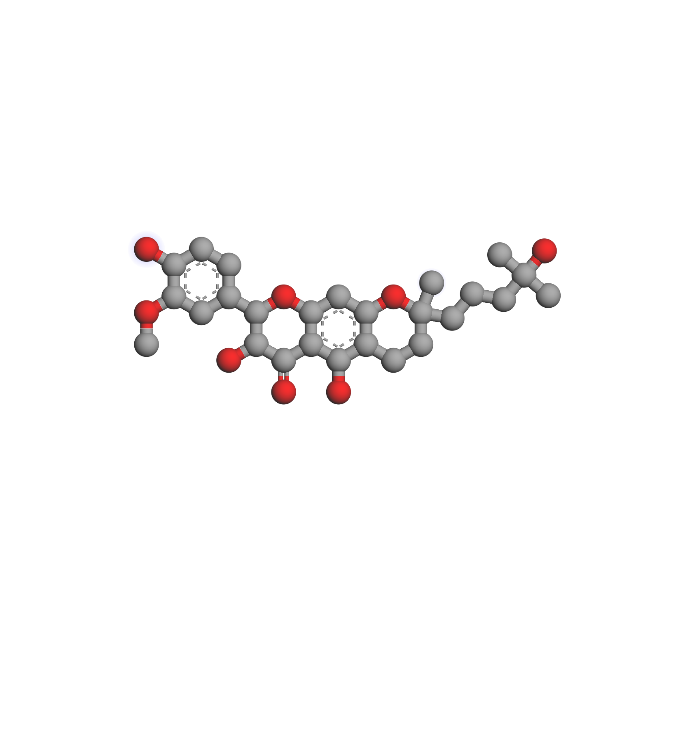 | -7.8 | **Hydrogen Bond:** Trp436 (5.29, 5.50)  **Carbon Hydrogen Bond:** Asn437 (4.42)  **Alkyl:** Lys440 (3.97), Leu368 (3.93, 4.79), Leu371 (5.33)  **π Alkyl:** Phe342 (4.22), Pro373 (6.12), Phe374 (6.32, 6.66, 6.78)  **π** **Cation:** Lys440 (4.93)  **van der Waals:** Phe338, Asp339, Asn343, Val367, Ser438, Asn439, Arg509 |
| 40 | 5280804 | Isoquercitrin | 464.4 | C_21_H_20_O_12_ | 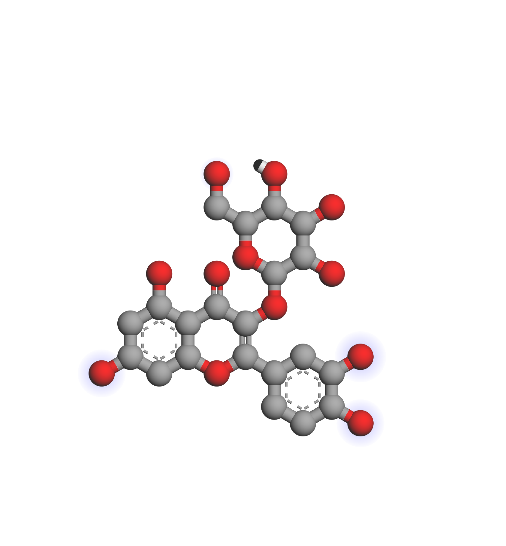 | -6.4 | **Hydrogen Bond:** Asn343 (3.50), Lys440 (3.70, 3.76, 4.20)  **Carbon Hydrogen Bond:** Ser438 (4.28)  **π Cation:** Lys440 (4.20, 4.69)  **van der Waals:** Phe342, Ala344, Trp436, Asn437, Asn439, Leu441, Arg509, Pro373, Phe375 |

| **Drug**  **likeness**  **Properties**  **Phytochemicals** | **MW (g/mol) (range ≤500 g)** | **Concensus Log Po/w (range ≤5)** | **No. of H-bond Acceptor (range ≤10)** | **No. of H-bond Donors (range ≤5)** | **Molar Refractivity (****range 40-130)** | **Lipinski** | **Veber** | **Bioavailability Score (range 0.4-0.6)** | **Synthetic accessibility (****range ≤6)** | **TPSA (****Å2) (range ≤100)** | **No of rotatable bonds (range 1-10)** | **Solubility (mg/ml)** |
| --- | --- | --- | --- | --- | --- | --- | --- | --- | --- | --- | --- | --- |
| Tomentin A | 442.50 | 3.59 | 7 | 4 | 120.61 | YES | YES | 0.55 | 4.56 | 116.45 | 5 | 8.63e-04 |
| Tomentin C | 486.55 | 3.97 | 8 | 3 | 131.57 | YES | YES | 0.55 | 4.88 | 114.68 | 7 | 1.56e-04 |
| Hyperoside | 464.38 | -0.25 | 12 | 8 | 110.16 | NO | NO | 0.17 | 5.32 | 210.51 | 4 | 1.43e+01 |
| Catechin gallate | 442.37 | 1.25 | 10 | 7 | 110.04 | YES | NO | 0.55 | 4.16 | 177.14 | 4 | 3.60e-01 |
| Corylifol A | 390.47 | 5.15 | 4 | 2 | 119.25 | YES | YES | 0.55 | 3.86 | 70.67 | 6 | 1.34e-05 |

**Suppl. Table 2**. Calculated ADME properties of the top five flavonoid compounds.

**Suppl. Table 3.** Predicted toxicity features of the top-ranked compounds using ProTox-II.

| S.No. | Classification | Property/Description | Tomentin A | Tomentin C | Hyperoside | Catechin gallate | Corylifol A |
| --- | --- | --- | --- | --- | --- | --- | --- |
| 1 | Prediction Accuracy | ---- | 69.26% | 69.26% | 72.9% | 100% | 68.07% |
| 2 | Predicted Toxicity Class (1-6) | Class 1: Most toxic, Class 6: Least toxic | 4 | 4 | 5 | 4 | 5 |
| 3 | Predicted LD50 (mg/kg) | --- | 2000 | 2000 | 5000 | 1000 | 2500 |
| 4 | Organ toxicity | Hepatotoxicity | Inactive  (0.79) | Inactive  (0.83) | Inactive  (0.82) | Inactive  (0.70) | Inactive  (0.75) |
| 5  6  7  8 | Toxicity end points | Carcinogenicity  Immunotoxicity  Mutagenicity  Cytotoxicity | Inactive  (0.61)  Active  (0.99)  Inactive  (0.63)  Inactive  (0.85) | Inactive  (0.66)  Active  (0.99)  Inactive  (0.63)  Inactive  (0.79) | Inactive  (0.85)  Active  (0.66)  Inactive  (0.76)  Inactive  (0.69) | Inactive  (0.54)  Inactive  (0.87)  Inactive  (0.70)  Inactive  (0.82) | Inactive  (0.59)  Active  (0.95)  Inactive  (0.70)  Inactive  (0.89) |
| 9  10  11  12  13  14  15 | Tox21-Nuclear receptor signalling pathways | Aryl hydrocarbon Receptor (AhR)  Androgen Receptor (AR)  Androgen Receptor Ligand Binding Domain (AR-LBD)  Aromatase  Estrogen Receptor Alpha (ER)  Estrogen Receptor Ligand Binding Domain (ER-LBD)  Peroxisome Proliferator Activated Receptor Gamma (PPAR-Gamma) | Inactive  (0.87)  Inactive  (0.89)  Inactive  (0.85)  Inactive  (0.69)  Inactive  (0.69)  Inactive  (0.90)  Inactive  (0.80) | Inactive  (0.88)  Inactive  (0.91)  Inactive  (0.83)  Inactive  (0.64)  Inactive  (0.76)  Inactive  (0.91)  Inactive  (0.84) | Inactive  (0.92)  Inactive  (0.90)  Inactive  (0.98)  Inactive  (1.0)  Inactive  (0.91)  Inactive  (0.99)  Inactive  (0.99) | Inactive  (0.85)  Inactive  (0.96)  Inactive  (0.95)  Inactive  (0.98)  Inactive  (0.87)  Inactive  (0.89)  Inactive  (0.92) | Inactive  (0.64)  Inactive  (0.96)  Inactive  (0.95)  Inactive  (0.62)  Inactive  (0.66)  Inactive  (0.79)  Inactive  (0.83) |
| 16  17  18  19  20 | Tox21-Stress response pathways | Nuclear factor (erythroid-derived 2)-like 2/antioxidant responsive element (nrf2/ARE)  Heat shock factor response element (HSE)  Mitochondrial Membrane Potential (MMP)  Phosphoprotein (Tumor Suppressor) p53  ATPase family AAA domain-containing protein 5 (ATAD5) | Inactive  (0.81)  Inactive  (0.81)  Active  (0.64)  Inactive  (0.55)  Inactive  (0.90) | Inactive  (0.84)  Inactive  (0.84)  Active  (0.64)  Inactive  (0.50)  Inactive  (0.90) | Inactive  (0.98)  Inactive  (0.50)  Inactive  (0.98)  Active  (0.50)  Inactive  (1.0) | Inactive  (0.98)  Inactive  (0.98)  Inactive  (0.79)  Inactive  (0.93)  Inactive  (0.97) | Inactive  (0.73)  Inactive  (0.73)  Active  (0.70)  Inactive  (0.64)  Inactive  (0.94) |

**Suppl. Table 4**. Comparison of MD simulation results on 200 ns for top five hits

| Molecules  MD analysis | Tomentin A | Tomentin C | Hyperoside | Catechin gallate | Corylifol A |
| --- | --- | --- | --- | --- | --- |
| RMSD (nm) | 0.27 | 0.18 | 0.17 | 0.14 | 0.23 |
| RMSF (nm) | 0.13 | 0.12 | 0.09 | 0.08 | 0.10 |
| Rg (nm) | 1.85 | 1.85 | 1.85 | 1.84 | 1.84 |
| SASA (nm^2^) | 113.60 | 113.33 | 113.55 | 111.58 | 115.61 |

**Suppl. Table 5**. List of calculated post MD interactions.

| **Compound** | **Interacting Pairs** | **Distance (Angstrom)** | **Type** | **Category** |
| --- | --- | --- | --- | --- |
| Corylifol A | A:SER496:HN - B:UNL528:O2 | 1.70 | Hydrogen Bond | Conventional Hydrogen Bond |
|  | B:UNL528:H1 - A:HIS505:O | 1.91 | Hydrogen Bond | Conventional Hydrogen Bond |
|  | A:ARG403:NH1 - B:UNL528 | 3.48 | Electrostatic | Pi-Cation |
|  | A:ARG403:NH2 - B:UNL528 | 3.66 | Electrostatic | Pi-Cation |
|  | A:ASP405:OD1 - B:UNL528 | 4.98 | Electrostatic | Pi-Anion |
|  | A:SER496:HN - B:UNL528 | 2.59 | Hydrogen Bond | Pi-Donor Hydrogen Bond |
|  | A:SER496:OG - B:UNL528 | 2.99 | Other | Pi-Lone Pair |
|  | A:TYR501 - B:UNL528 | 5.01 | Hydrophobic | Pi-Pi Stacked |
|  | A:TYR449 - B:UNL528 | 5.35 | Hydrophobic | Pi-Alkyl |
|  | B:UNL528 - A:ARG403 | 4.78 | Hydrophobic | Pi-Alkyl |
|  |  |  |  |  |
| Catechin gallate | X:THR393:HN - X:UNL528:O4 | 2.84 | Hydrogen Bond | Conventional Hydrogen Bond |
|  | X:THR393:HG1 - X:UNL528:O4 | 2.91 | Hydrogen Bond | Conventional Hydrogen Bond |
|  | X:ALA522:HN - X:UNL528:O1 | 1.95 | Hydrogen Bond | Conventional Hydrogen Bond |
|  | X:PRO521:HB1 - X:UNL528 | 2.55 | Hydrophobic | Pi-Sigma |
|  | X:ALA522:HB2 - X:UNL528 | 2.75 | Hydrophobic | Pi-Sigma |
|  | X:CYS391:SG - X:UNL528 | 4.00 | Other | Pi-Sulfur |
|  | X:CYS525:SG - X:UNL528 | 3.75 | Other | Pi-Sulfur |
|  | X:CYS391 - X:UNL528 | 5.09 | Hydrophobic | Alkyl |
|  | X:ALA522 - X:UNL528 | 4.21 | Hydrophobic | Alkyl |
|  | X:UNL528 - X:CYS391 | 4.66 | Hydrophobic | Pi-Alkyl |
|  | X:UNL528 - X:LEU518 | 4.84 | Hydrophobic | Pi-Alkyl |
|  |  |  |  |  |
| Hyperoside | B:UNL528:H8 - A:SER494:OG | 1.92 | Hydrogen Bond | Conventional Hydrogen Bond |
|  | A:ARG493:HD2 - B:UNL528:O1 | 2.55 | Hydrogen Bond | Carbon Hydrogen Bond |
|  | B:UNL528:H03 - B:UNL528:O2 | 2.74 | Hydrogen Bond | Carbon Hydrogen Bond |
|  | A:PHE490 - B:UNL528 | 4.83 | Hydrophobic | Pi-Pi Stacked |
|  |  |  |  |  |
| Tomentin A | A:CYS488:HN - B:UNL528:O7 | 2.25 | Hydrogen Bond | Conventional Hydrogen Bond |
|  | B:UNL528:H2 - B:UNL528:O5 | 2.14 | Hydrogen Bond | Conventional Hydrogen Bond |
|  | B:UNL528:H3 - A:ASN481:O | 2.68 | Hydrogen Bond | Conventional Hydrogen Bond |
|  | B:UNL528:H3 - A:VAL483:O | 2.15 | Hydrogen Bond | Conventional Hydrogen Bond |
|  | B:UNL528:H4 - A:PHE486:O | 1.91 | Hydrogen Bond | Conventional Hydrogen Bond |
|  | A:PHE490 - B:UNL528 | 4.21 | Hydrophobic | Pi-Pi Stacked |
|  | B:UNL528 - A:LEU452 | 5.26 | Hydrophobic | Alkyl |
|  | A:PHE490 - B:UNL528 | 5.34 | Hydrophobic | Pi-Alkyl |
|  | B:UNL528 - A:ALA484 | 5.02 | Hydrophobic | Pi-Alkyl |
|  |  |  |  |  |
| Tomentin C | A:GLY502:HN - B:UNL528:O7 | 1.95 | Hydrogen Bond | Conventional Hydrogen Bond |
|  | A:TYR501 - B:UNL528:C26 | 4.89 | Hydrophobic | Pi-Alkyl |
